# Upregulated hsa_circRNA_100269 inhibits the growth and metastasis of gastric cancer through inactivating PI3K/Akt axis

**DOI:** 10.1371/journal.pone.0250603

**Published:** 2021-04-26

**Authors:** Zhongli Wang, Chao Liu

**Affiliations:** 1 Department of General Surgery, the First Affiliated Hospital of Jinzhou Medical University, Jinzhou, Liaoning, P.R. China; 2 Department of Developmental Biology, Jinzhou Medical University, Jinzhou, Liaoning, P.R. China; Chung Shan Medical University, TAIWAN

## Abstract

The pathogenesis of GC involves the complex networking of multiple signaling pathways; however, the detailed mechanisms of tumorigenesis of GC remains largely unknown. Therefore, it is necessary to explore novel diagnostic/prognostic biomarkers for GC. In this study, the levels of hsa_circRNA_100269 in gastric cancer (GC) samples and cells were examined, and its effects on the biological functions of GC cells were elucidated. The levels of hsa_circRNA_100269 in specimens/cell lines were examined using RT-qPCR. Cell models with hsa_circRNA_100269 overexpression or knockdown were generated using lentiviral vectors. Cell viability was determined by MTT assay; cell migratory/invasive activity was evaluated using wound healing/Transwell assay. Cell cycle arrest and apoptosis were assessed by flow cytometry; expression of associated markers involved in cell apoptosis, EMT and the PI3K/Akt signaling were determined by RT-qPCR/immunoblotting. In vivo study was also performed using hsa_circRNA_100269 knockout mice. Our findings revealed downregulation of hsa_circRNA_100269 in GC tissues compared to non-cancerous control. Additionally, the levels of PI3K were remarkably elevated in GC tissues, where hsa_circRNA_100269 and PI3K was negatively correlated. Moreover, the expression of hsa_circRNA_100269 was associated with histology grade and occurrence of metastasis in GC patients. In addition, hsa_circRNA_100269 was downregulated in GC cells compared to normal gastric epithelial cells. Overexpressed hsa_circRNA_100269 notably inhibited the proliferation, migration, invasion and EMT of GC cells, whereas cell cycle arrest at G0/G1 phase was promoted and cell apoptosis was enhanced. Moreover, the PI3K/Akt signaling was involved in hsa_circRNA_100269-regulated GC cell proliferation, migration, invasion, EMT and apoptosis. Knockdown of hsa_circRNA_100269 also remarkably induced tumor growth in mouse model. In summary, our findings indicated that the levels of hsa_circRNA_100269 were reduced in GC. Furthermore, hsa_circRNA_100269 could suppress the development of GC by inactivating the PI3K/Akt pathway. More importantly, hsa_circRNA_100269/PI3K/Akt axis may be a novel therapeutic candidate for GC treatment.

## Introduction

Gastric cancer (GC) is a type of aggressive tumor and one leading cause of cancer-related mortality, and the global incidence of GC are rising [1]. The pathogenesis of GC is complicate, and a previous report has revealed that the five-year survival rate of GC patients in China remains poor [~25–30%; 2]. Modern therapeutic strategies for GC are developed and endoscopic and surgical resection are widely used for GC patients, but the prognosis of GC is still poor. The therapeutic outcome of patients with GC is relevant to disease staging at diagnosis, and the mortality of patients with advanced GC remains high [~30–50%; 3–5]. Nevertheless, the detailed mechanisms during the onset and development of GC are still unclear. So it is urgent to identify putative prognostic biomarkers for GC.

Circular RNAs (circRNAs) are a group of new non-coding RNAs. Not like their linear counterparts, they can form a continuous circle which is characterized with a more stable structure [6–8]. Due to the absence of free 5’- or 3’- overhangs in the structure, circRNAs are resistant to the degradation caused by exonuclease [6]. Furthermore, the distributions of circRNAs are tissue specific [8]. Some circRNAs have been revealed as putative gene regulators, the regulatory functions of most circRNAs are not fully understood [9]. Statistical estimates and biochemical assays provided strong evidence that a substantial fraction of the spliced transcripts from hundreds of genes are circular RNAs [7]. A previous study also indicated that over one-third of abundant circRNAs is dynamically regulated by the alternative splicing factor, Quaking (QKI), which itself is regulated during EMT [8].

Recent studies have revealed that circRNAs could be able to exert their function as miRNA ‘sponges’ which competitively suppress the activity of corresponding miRNAs [10]. Moreover, ciRS-7 contains more than 70 selectively conserved miRNA target sites, and it is highly and widely associated with Argonaute (AGO) proteins in a miR-7-dependent manner [10]. In addition, circRNAs are involved in the initiation and development of numerous diseases, including nervous system disorders and tumour [11–13]. CircRNAs are involved in tumor progression of numerous types of cancer by regulating tumor cell growth through various signaling, e.g. via targeting corresponding miRNAs. For instance, antisense to the cerebellar degeneration-related protein 1 transcript (CDR1as), is densely bound by miRNA effector complexes and harbours 63 conserved binding sites for the ancient miRNA miR-7 [6]. Furthermore, a previous research has revealed the regulatory functions of a novel circRNA SFMBT2 in GC and it could act as a sponge for miR-182-5p, which promotes the growth of GC cells through the upregulation of CREB [14]. But the effects of the majority of circRNAs during tumorigenesis remain unclear. Furthermore, recent report revealed downregulation of hsa_circRNA_100269 in GC, which inhibits the proliferation of tumor cells through targeting miR-630 [15]. These findings have highlighted the importance of hsa_circRNA_100269/miR-630 axis during the development of GC. However, the detailed functions and the exact molecular mechanisms of the hsa_circRNA_100269/miR-630-modulated signaling in GC have not been reported. Therefore, the regulatory roles and novel molecular targets of hsa_circRNA_100269/miR-630 in GC were still elusive and require further elucidation.

Previous studies have suggested that miR-630 is associated with the regulation of various biological processes including EMT through numerous pathways such as the PI3K/Akt signaling [16,17]. For example, the extract of herbal medicine celastrus orbiculatus is able to induce cell apoptosis and autophagy by suppressing the PI3K/Akt/mTOR pathway [18]. In addition, miR-630 was able to suppress EMT of GC cells via FoxM1, supposedly via the inactivation of the Ras/PI3K/Akt pathway [16]. PI3K can be activated by oncogenes, then activated PI3K is capable of promoting the growth and metastasis of tumour cells [18]. Akt is a downstream molecule of PI3K, and it is involved in various biological processes, including proliferation, invasion and EMT [19]. Moreover, gankyrin activates the PI3K/GSK-3beta/beta-catenin signaling and promotes the aggressiveness and progression of tumor [19]. Activation of Akt may induce the migration, invasion and EMT of cancer cells [20]. Furthermore, salidroside triggers the apoptosis and autophagy of osteosarcoma cells by inactivating the PI3K/Akt/mTOR pathway [20]. Activated PI3K/Akt axis leads to enhanced migratory/invasive activity of human osteosarcoma cells [21]. Thus, inhibition of the PI3K/Akt pathway could be a promising therapeutic strategy for cancer treatment. In this study, the influences of the hsa_circRNA_100269-regulated PI3K/Akt pathway on growth/metastasis in GC cells were elucidated. Our data indicated that cell cycle arrest was induced at G0/G1 phase and cell apoptosis was enhanced in GC cells with hsa_circRNA_100269 overexpression, where the levels of p-Akt and Bcl-2 were also notably reduced. Our results revealed that overexpression of hsa_circRNA_100269 suppressed the proliferation, migration and invasion and promoted cell cycle arrest and apoptosis in GC cells by targeting PI3K/Akt axis.

## Materials and methods

### Clinical samples

56 matched GC and non-tumour samples were collected during surgery at the First Affiliated Hospital of Jinzhou Medical University from June 2017-July 2018. The tissues were sectioned and snap-frozen using liquid nitrogen post-surgery, and stored at -80˚C. The patients were divided into hsa_circRNA_100269 high- or low-expression group according to the mean hsa_circRNA_100269 expression value. The clinic-pathological features of recruited patients were listed in Table 1. Overall survival rates were analysed using Kaplan-Meier method. Written informed consents were signed by the patients, and all the samples were anonymized. The present study was approved by the Ethics Committee of the First Affiliated Hospital of Jinzhou Medical University.

**Table 1:** Clinical and pathological characteristics of patients with GC enrolled in the present study.

Parameters	n	hsa_circRNA_100269 expression		P value
		Low	High	
Gender				0.464
Male	26	12	14	
Female	30	16	14	
Age (years)				0.429
>60	24	11	13	
≤60	32	17	15	
Tumor size (cm)				0.524
>5	24	12	12	
≤5	32	16	16	
T stage				0.401
T4a/4b	33	18	15	
T3 or lower	23	10	13	
Histology grade				0.023*
I-II	34	13	21	
III-IV	22	15	7	
Smoking				0.485
Yes	31	16	15	
No	25	12	13	
Metastasis				0.019*
Yes	22	15	7	
No	34	13	21	

Differences among variable were analyzed using the χ2 test

* indicates that the values have statistically significant differences.

### Cell culture

Four human GC cell lines (AGS, NCI-N87 and MKN-45) and one normal human gastric epithelial cell line (GES-1) were obtained from the American Type Culture Collection (Manassas, VA, USA). The cells were maintained using DMEM containing 10% fetal bovine serum (FBS), 100 μg/ml streptomycin and 100 U/ml penicillin (HyClone; GE Healthcare Life Science), and cultured at 37˚C in a incubator supplied with 5% CO_2_.

### Cell transfection

In order to establish the cell model overexpressing hsa_circRNA_100269, wildtype (o/e-hsa_circRNA_100269) or mutant (o/e-NC) hsa_circRNA_100269 sequence was amplified using PCR, then subcloned into pcDNA3.1 His C vector (Invitrogen; Thermo Fisher Scientific, Inc., Waltham, MA, USA). In hsa_circRNA_100269 knockdown model, shRNA sequences against hsa_circRNA_100269 (sh-hsa_circRNA_100269) or negative control (sh-NC) were obtained from Genepharm Co. Ltd. (Shanghai, China). Following annealing, shRNA were inserted in lentiviral pU6-Luc-Puro vector (Genepharm Co. Ltd.). Cells without any shRNA treatment were used as the control group. Up- or downregulation of hsa_circRNA_100269 was examined by RT-qPCR. All the transfections were performed using Lipofectamine^®^2000 (Invitrogen; Thermo Fisher Scientific, Inc.). Eight hours following transfection, culture media were replenished with fresh DMEM supplemented with 10% FBS. For the inhibition of PI3K signalling, cells were treated with LY294002 (10μM; Cell Signaling Technology, Beverly, USA).

### RNA extraction and reverse transcription-quantitative polymerase chain reaction (RT-qPCR)

Total RNA from clinical samples or cells was extracted by TRIzol^®^ reagent (Invitrogen; Thermo Fisher Scientific, Inc.). Quality of isolated RNA was evaluated using Bioanalyzer (2100; Agilent). RNA was then reverse transcribed into cDNA by a PrimeScript™ RT kit (Takara Biotechnology Co., Ltd., Dalian, China). The target cDNA was amplified using SYBR Green PCR Master Mix (TaKaRa Biotechnology Co., Ltd.), which was carried out using an ABI 7500 Real-Time PCR system (Thermo Fisher Scientific, Inc.) Endogenous GAPDH was used as internal control. The sequences of forward and reverse primer were as follows: hsa_circRNA_100269, 5’-CTAACTATGGTCGGACGGATGA-3’ and 5’-CAATGATAAACCACAGACTTCGC-3’; PI3K, 5’-AACACAGAAGACCAATACTC-3’ and 5’-TTCGCCATCTACCACTAC-3’; E-cad, 5’-AAGAAGCTGGCTGACATGTACGGA-3’ and 5’-CCACCAGCAACGTGATTTCTGCAT-3’; vimentin, 5’-AGAACCTGCAGGAGGCAGAAGAAT-3’ and 5’-TTCCATTTCACGCATCTGGCGTT-3’; snail, 5’-TTTCTGGTTCTGTGTCCTCTGCCT-3’ and 5’-TGAGTCTGTCAGCCTTTGTCCTGT-3’; Bax, 5’-TAATCCCAGCGCTTTGGAA-3’ and 5’- TGCAGAGACCTGGATCTAGCAA-3’; cas-9, 5’-CATTTCATGGTGGAGGTGAAG-3’ and 5’-GGGAACTGCAGGTGGCTG-3’; MMP9, 5’-CAGAGATGCGTGGAGAGT-3’ and 5’-TCTTCCGAGTAGTTTTGG-3’; GAPDH, 5’-GCAAGAGCACAAGAGGAAGA-3′ and 5’-ACTGTGAGGAGGGGAGATTC-3’. PCR program was 95˚C for 5 min, followed by 45 cycles of 95˚C for 15s, 60˚C for 20s and 72˚C for 10s.

### Western blot analysis

Total protein was extracted by radioimmunoprecipitation assay buffer (Beyotime Institute of Biotechnology, Shanghai, China). The concentration of extracted protein was determined using bicinchoninic acid assay (Beyotime Institute of Biotechnology). Equal amount (30 μg) of samples were loaded on SDS-PAGE gel and subsequently transferred onto a PVDF membrane (EMD Millipore, Billerica, MA, USA). Membranes were blocked using tris-buffered saline (TBS) with 5% skimmed milk at room temperature for 2 h and incubated using correspondent primary antibodies: PI3K (1:2000; cat. no. ab140307; abcam), Akt (1:1000; cat. no. 9272; Cell Signaling Technology), p-Akt^S473^ (1:1000; cat. no. 4058; Cell Signaling Technology), p53 (1:1000, cat. no. 9282; Cell Signaling Technology), Bcl-2 (1:1000, cat. no. 15071; Cell Signaling Technology), cyclin D1 (1:2000, cat. no. 2926; Cell Signaling Technology), E-cad (1:1000, cat. no. 3195; Cell Signaling Technology), vimentin (1:2000; cat. no. 5741; Cell Signaling Technology), snail (1:1000; cat. no. 3879; Cell Signaling Technology), Bax (1:1000; cat. no. 2772; Cell Signaling Technology), cas-9 (1:2000; cat. no. 14697; Cell Signaling Technology), MMP9 (1:1000; cat. no. 3852; Cell Signaling Technology) or GAPDH (1:1,000; cat. no. sc-47724; Santa Cruz Biotechnology Inc.) at 4˚C overnight. The membranes were subsequently incubated with corresponding horseradish peroxidase-conjugated anti-mouse (1:5,000; cat. no. sc-2371; Santa Cruz Biotechnology Inc.) or anti-rabbit IgG (1:5000; cat. no. sc-2357; Santa Cruz Biotechnology Inc.) at room temperature for 1h. Protein bands were visualized by an enhanced ECL protein detection kit (Pierce Biotechnology; Thermo Fisher Scientific, Inc). Signals were quantified using densitometric method by Image J software (NIH, Bethesda, MD, USA).

### Cell proliferation

Cells were harvested 24 h post-transfection, and 1x10^4^ cells were placed in 96-well plates. The proliferation of cells was examined using MTT assay (Sigma-Aldrich; Merck KGaA) at day 1, 2, 3 and 4. Briefly, 20 μl of MTT solution was added into each well and incubated at 37˚C for 4 h, the absorbance at 450 nm was detected using a microplate reader (Bio-Rad Laboratories, Inc., Hercules, CA, USA).

### Wound healing

Cells were seeded onto 6-well plates at a density of 4x10^5^ cells/well and transfected with corresponding vectors. After the cells reached the confluency of 80–100%, and they were pre-treated with 10ug/mL mitomycin C (Thermo Fisher Scientific) for two hours prior to wound healing assay. Then, cell monolayer was scratched in a straight line with a sterile micropipette tip and washed three times with PBS, which was replaced with fresh DMEM. Subsequently, the scratch width changes were observed immediately following the scratch and at 6, 12 and 24 h. The images were captured using a fluorescence microscope (magnificationx100, Olympus Corporation, Tokyo, Japan). The migration of cells was determined by ImageJ 6.0 using the following formula: Migration area ratio = proportion of closed wound area/entire field of view area.

### Transwell assay

Cells were pre-treated with 10ug/mL mitomycin C (Thermo Fisher Scientific) for two hours prior to assay. A total of 1x10^5^ cells were suspended using FBS-free culture medium and seeded onto the Matrigel^®^-pre-coated (Sigma-Aldrich, St. Louis, MO, USA) upper chamber (BD Biosciences, Franklin Lakes, New Jersey, USA). Subsequently, 500 μl of culture medium containing 10% FBS was added into the lower chamber. After overnight incubation, non-invasive cells were detached using a cotton swab, while invaded cells in the lower chamber were fixed using 4% paraformaldehyde and stained by 0.5% crystal violet. The numbers of invasive cells were counted in five randomly selected fields using an inverted microscope (magnificationx200, Olympus Corporation, Japan).

### Cell cycle and apoptosis

Cells were seeded onto 6-well plates at a density of 4x10^5^ cells per well following the treatments with o/e-hsa_circRNA_100269 or o/e-NC, respectively. Then, cells were collected using low-speed centrifugation (1000rpm) at 4˚C for 5 mins. Cell pellets were rinsed and re-suspended in PBS, subsequently fixed with 70% pre-chilled ethanol and stored at 4˚C for two days. Cells were lysed prior to flow cytometry, centrifuged and then re-suspended using propidium iodide (PI, Sigma-Aldrich, USA) staining buffer containing 50 μl/ml of PI with 250 μl/ml RNase A. Cell cycle distributions were determined by a flow cytometer (BD Biosciences, USA) and then analysed using Flowjo version 7.6 software (Flowjo LLC, USA). To evaluate cell apoptosis, the suspended cells was incubated in dark at 4˚C for 30 mins and stained with 5 μl annexin V-FITC (JingMei Biotech, Beijing, China), and apoptosis was examined using a a flow cytometer (BD Biosciences, USA) and subsequently analysed using Flowjo version 7.6 software (Flowjo LLC, USA).

### In vivo nude mouse xenograft

Doxycycline-inducible constructs were produced by inserting hsa_circRNA_100269 into a tet-on circRNA expression vector (Addgene #92351). The CMV promoter was replaced with a tet-on promoter to activate transcription in the presence of doxycycline (Dox). Genomic region of hsa_circRNA_100269 was amplified and cloned into the NheI/MluI-digested vector. The construct was then transfected into MNK-45 cells, which were further used for inoculation into nude mice. BALB/C nude mice were purchased from the Laboratory Animal Research Centre of Jinzhou Medical University. The mice were routinely housed in a temperature-controlled environment (22±2˚C) with 60% relative humidity, under a 12-h dark/light cycle with libitum access to food and water for at least three days before the experiments. Mice were randomly grouped (n = 5 in each group) and injected with MNK-45 cells. Briefly, a total of 1x107 cells were suspended in 200μl PBS and injected into the back subcutaneously. Mice with developing tumors were monitored four times a week. For the dox-induction group, the cells were pre-treated with 1 μg/mL doxycycline to prime circRNA expression one day before harvesting for injection. Dox-induction animals were given 1 mg/mL doxycycline water, which was changed every 2 days for the duration of the experiment. Six weeks post-injection, the mice were sacrificed, and the tumor tissues were removed and examined. Tumor volume was calculated as follows: V (mm^3^) = (length x width^2^)/2. To initiate metastasis, 1x10^5^ cells were suspended in 20 μl PBS and then injected in the lateral tail vein of mice. A total of five mice were included in each experimental group. The protocol of animal experiment was approved by the Ethics Committee of the First Affiliated Hospital of Jinzhou Medical University.

### Statistical analysis

Data were presented as means ± standard error of mean and analysed using SPSS 17.0 (SPSS, Inc., Chicago, IL, USA). The significance of differences was analysed using one-way analysis of variance (ANOVA) or the Student’s t-test. A student-Newman-Keuls test was carried out after ANOVA. The association between RNA expression was determined using Spearman’s correlation analysis. All the experiments were performed in triplicate. P<0.05 was considered to indicate a statistically significant difference.

## Results

### The expression of hsa_circRNA_100269 is decreased in GC

**Fig 1:**
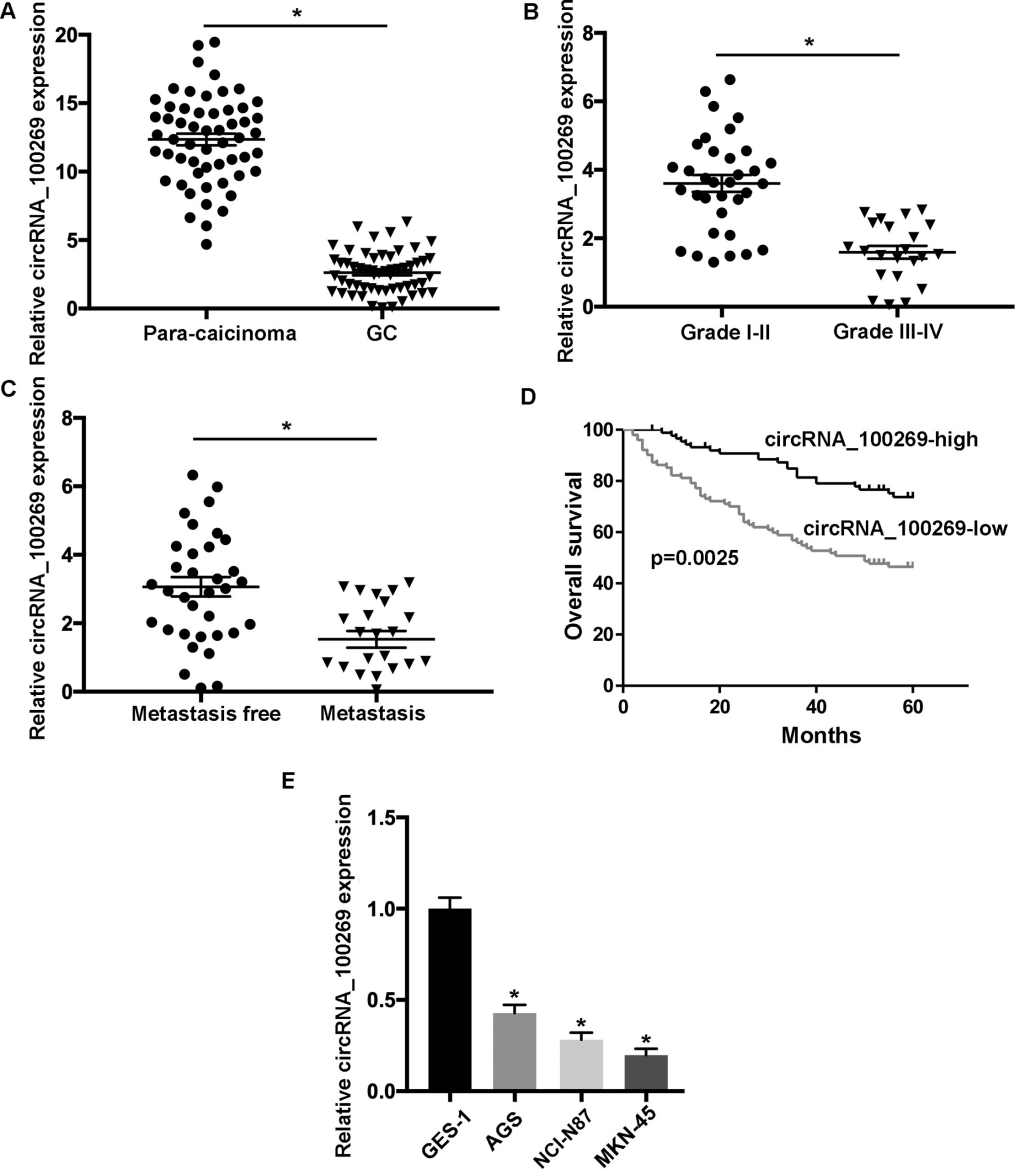
The expression of hsa_circRNA_100269 is downregulated in GC tissues and cells. (A) The level of hsa_circRNA_100269 was determined in 56 GC tissues and paired non-tumour samples using reverse transcription-quantitative polymerase chain reaction. (B) Hsa_circRNA_100269 expression was assessed in GC patients with different tumour grades. (C) The level of hsa_circRNA_100269 was evaluated in GC tissues with or without metastasis. (D) Survival analysis of GC patients with low- or high- hsa_circRNA_100269 expression. (E) The expression of hsa_circRNA_100269 was examined in GC cell lines (AGS, NCI-N87 and MKN-45) and one normal human gastric epithelial cell line (GES-1). All the experiments were performed in triplicate. *P<0.05 vs. corresponding control group. GC, gastric cancer.

The levels of hsa_circRNA_100269 were examined in 56 matched GC and non-tumour samples by RT-qPCR. The data suggested that hsa_circRNA_100269 was significantly downregulated in GC samples compared to para-carcinoma controls (Fig 1A). Additionally, the relationship between hsa_circRNA_100269 and the development of GC was evaluated, and the results indicated that hsa_circRNA_100269 was notably reduced in patients with advanced GC (Fig 1B). Additionally, the expression of hsa_circRNA_100269 was remarkably downregulated in GC patients with metastasis (Fig 1C). Moreover, GC patients with relatively low levels of hsa_circRNA_100269 exhibited significantly reduced overall survival (p = 0.0025, log-rank test; Fig 1D). Furthermore, the results of Cox regression revealed that tumor size, T stage, histology grade, metastasis and circrRNA_100269 expression were independent risk factors to predict the prognosis of GC patients (Table 2). Similarly, downregulation of hsa_circRNA_100269 was also detected in GC cells comparing to normal gastric epithelial cells (Fig 1E). Taken all together, the level of hsa_circRNA_100269 was decreased in GC, which may lead to tumor progression.

**Table 2:** Influence factors of the prognosis in GC patients were identified.

Parameters	χ2	P value	95%CI
Tumor size	10.263	P<0.05	2.054~3.784
T stage	11.352	P<0.05	3.216~6.423
Histology grade	12.639	P<0.05	2.534~11.332
Metastasis	14.826	P<0.05	3.840~13.618
CircRNA_100269 expression	12.304	P<0.05	1.891~8.453

Cox regression analysis was used to analyse the influence factors of patient prognosis.

### Overexpression of hsa_circRNA_100269 suppresses the growth, metastasis and EMT of GC cells

**Fig 2:**
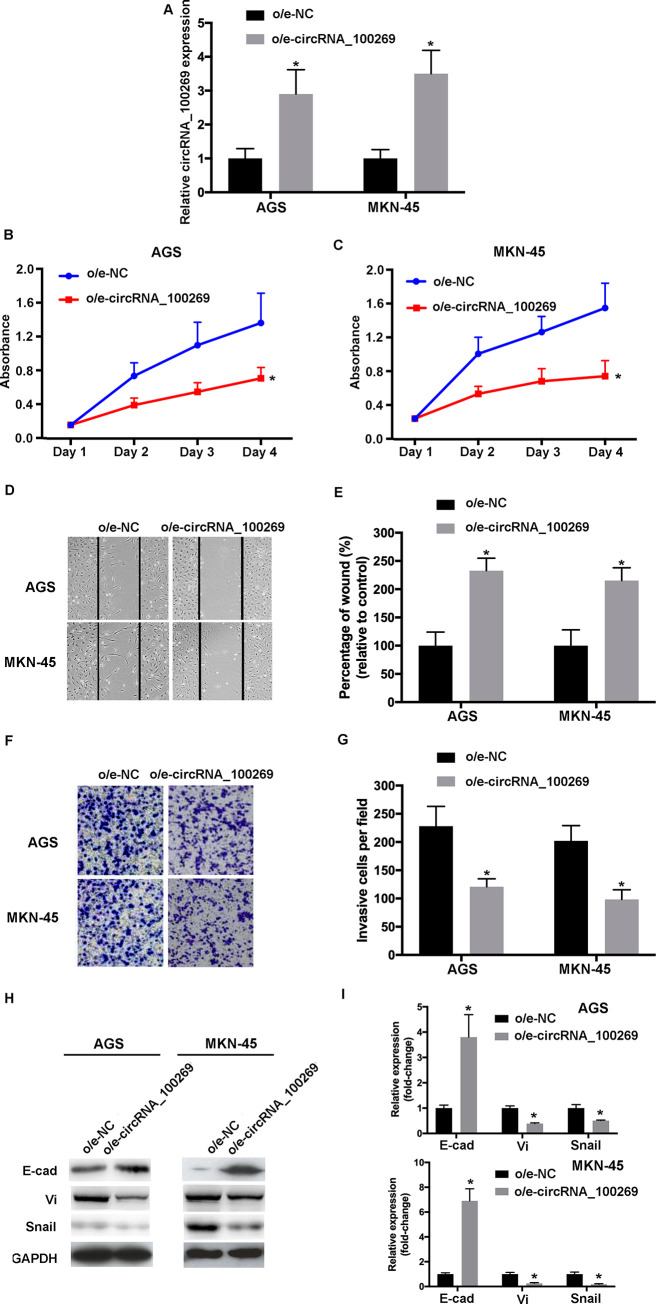
Upregulated hsa_circRNA_100269 inhibits the proliferation, migration, invasion and EMT of GC cells. (A) Transfection efficiency of o/e-hsa_circRNA_100269 was confirmed using RT-qPCR. (B and C) The proliferation of GC cells transfected with o/e-hsa_circRNA_100269 or o/e-NC were determined using Cell Counting Kit-8 assay. (D and E) The migration of transfected AGS and MKN-45 cells were evaluated using a wound healing assay (magnificationx100). (F and G) The invasive activity of GC cells transfected with o/e-hsa_circRNA_100269 or o/e-NC were examined (magnificationx200). (H and I) The expression levels of EMT-associated markers were evaluated using RT-qPCR and western blotting. All the experiments were performed in triplicate. *P<0.05 vs. o/e-NC. GC, gastric cancer; NC, negative control; RT-qPCR, reverse transcription-quantitative polymerase chain reaction.

In order to investigate the effects of hsa_circRNA_100269 on the progression of GC, hsa_circRNA_100269 was overexpressed in AGS and MKN-45 cells. The transfection efficiencies were evaluated by RT-qPCR (Fig 2A). Furthermore, the data of MTT assay revealed that the viabilities of GC cells transfected with o/e-hsa_circRNA_100269 was suppressed (Fig 2B and 2C). Would healing assay was performed to evaluate cell migration, whereas invasive activity was determined using transwell assay. Our data revealed that cell migration/invasion was inhibited within the hsa_circRNA_100269 overexpression group compared with the control (Fig 2D–2G). To investigate the effects of overexpressed hsa_circRNA_100269 on EMT of GC cells, the protein and mRNA levels of associated molecules such as E-cad, vimentin and snail were evaluated. The expression of abovementioned proteins were affected in cells transfected with o/e- hsa_circRNA_100269 (Fig 2H and 2I). In summary, overexpressed hsa_circRNA_100269 may result in suppressed proliferation, migration, invasion and EMT of GC cells.

### Overexpressed hsa_circRNA_100269 enhances cell cycle arrest and apoptosis in GC cells

**Fig 3:**
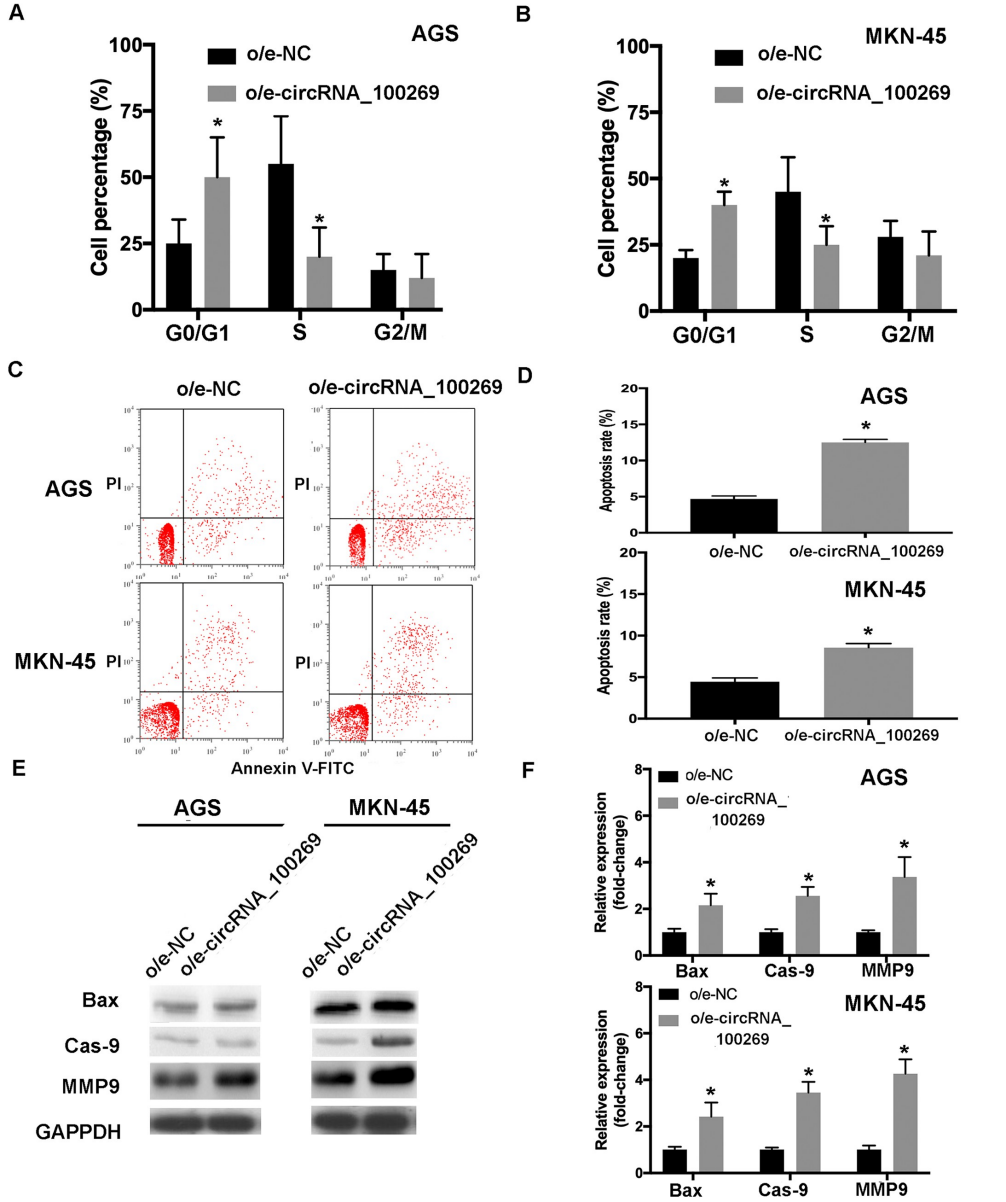
Overexpression of hsa_circRNA_100269 induces cell cycle arrest and apoptosis in GC cells. (A and B) The distribution of cell cycle and apoptosis in GC cells overexpressing hsa_circRNA_100269 were examined. (C and D) The apoptosis of AGS and MKN-45 cells transfected with o/e-hsa_circRNA_100269 were also determined using flow cytometry. (E and F) The expression levels of apoptosis-associated markers were evaluated in transfected GC cells compared with the control. All the experiments were performed in triplicate. *P<0.05 vs. o/e-NC. GC, gastric cancer.

According to the abovementioned results, hsa_circRNA_100269 was able to affect the proliferation and metastasis of GC cells. Furthermore, to investigate the influences of overexpressed hsa_circRNA_100269, the distribution of cell cycle and apoptosis in GC cells transfected with o/e-hsa_circRNA_100269 were evaluated compared with the control. The results revealed that GC cell cycle was dramatically shifted from S and G2/M phase to G0/G1 phase, and the cell percentage in G0/G1 phase was significantly increased, whereas those in S phase was notably reduced (Fig 3A and 3B). Furthermore, the results of flow cytometry revealed that overexpressed hsa_circRNA_100269 also promoted the apoptosis of GC cells (Fig 3C and 3D), which was confirmed by the upregulation of apoptosis-related markers including Bax, Cas-9 and MMP9 (Fig 3E and 3F). Our findings suggested that upregulation of hsa_circRNA_100269 could arrest cell cycle in G0/G1 phase to induce cell apoptosis.

### Knockdown of hsa_circRNA_100269 induces the development of GC cells

**Fig 4:**
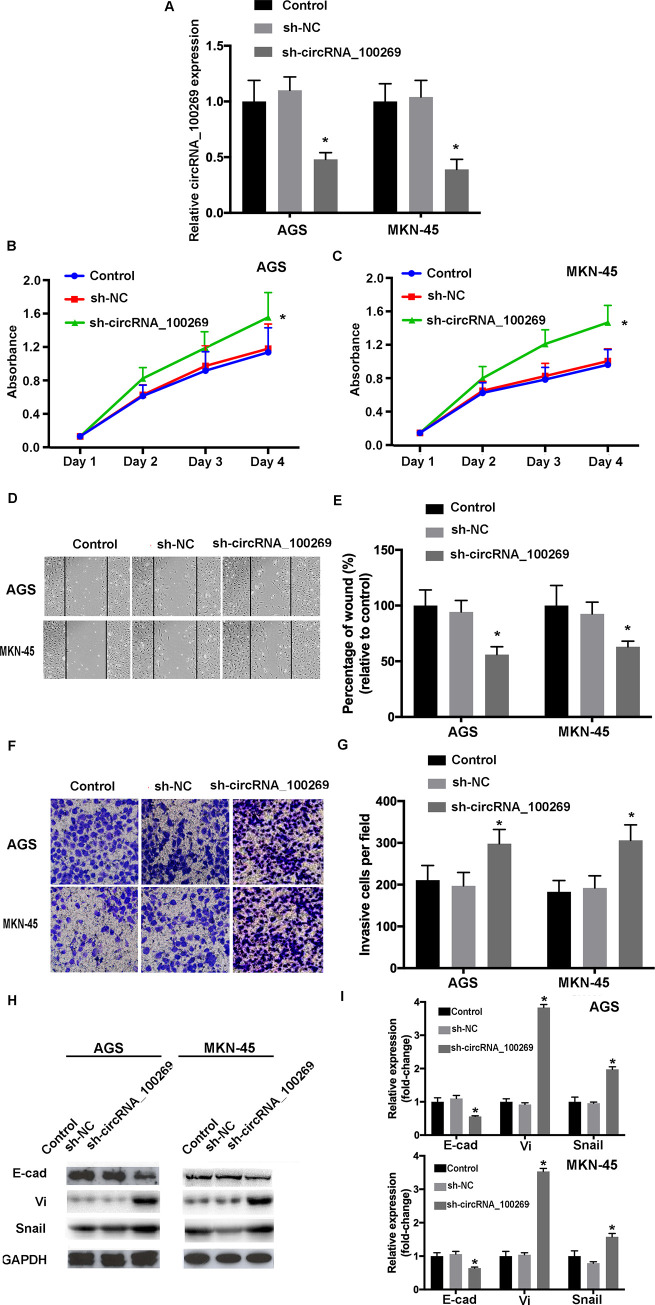
Knockdown of hsa_circRNA_100269 promotes the proliferation, migration, invasion and EMT of GC cells. (A) Transfection efficiency of sh-hsa_circRNA_100269 was evaluated by RT-qPCR. (B and C) The viabilities of GC cells transfected with sh-hsa_circRNA_100269 or sh-NC were examined using Cell Counting Kit-8 assay. (D-G) The migration and invasion of transfected AGS and MKN-45 cells were determined using wound healing (magnificationx100) and Transwell assay (magnificationx200). (H and I) The levels of EMT-related molecules were determined using RT-qPCR and western blotting. All the experiments were performed in triplicate. *P<0.05 vs. non-transfected cells. GC, gastric cancer; NC, negative control; RT-qPCR, reverse transcription-quantitative polymerase chain reaction.

As part of gain- and loss-of-function study, hsa_circRNA_100269 was knockdown in GC cells. The efficiencies of shRNA were confirmed by RT-qPCR (Fig 4A). MTT assay indicated that the cell viabilities were increased following the transfection with sh- hsa_circRNA_100269 (Fig 4B and 4C). The results of wound healing and transwell assays revealed that cell migrating and invasive abilities were enhanced in GC cells transfected with sh-hsa_circRNA_100269 (Fig 4D–4G). Furthermore, the levels of EMT-related molecules were influenced in sh-hsa_circRNA_100269 transfected group (Fig 4H and 4I). Taken all together, knockdown of hsa_circRNA_100269 could lead to enhanced proliferation, migration, invasion and EMT within GC cells.

### Knockdown of hsa_circRNA_100269 suppressed cell cycle arrest and apoptosis in GC cells

**Fig 5:**
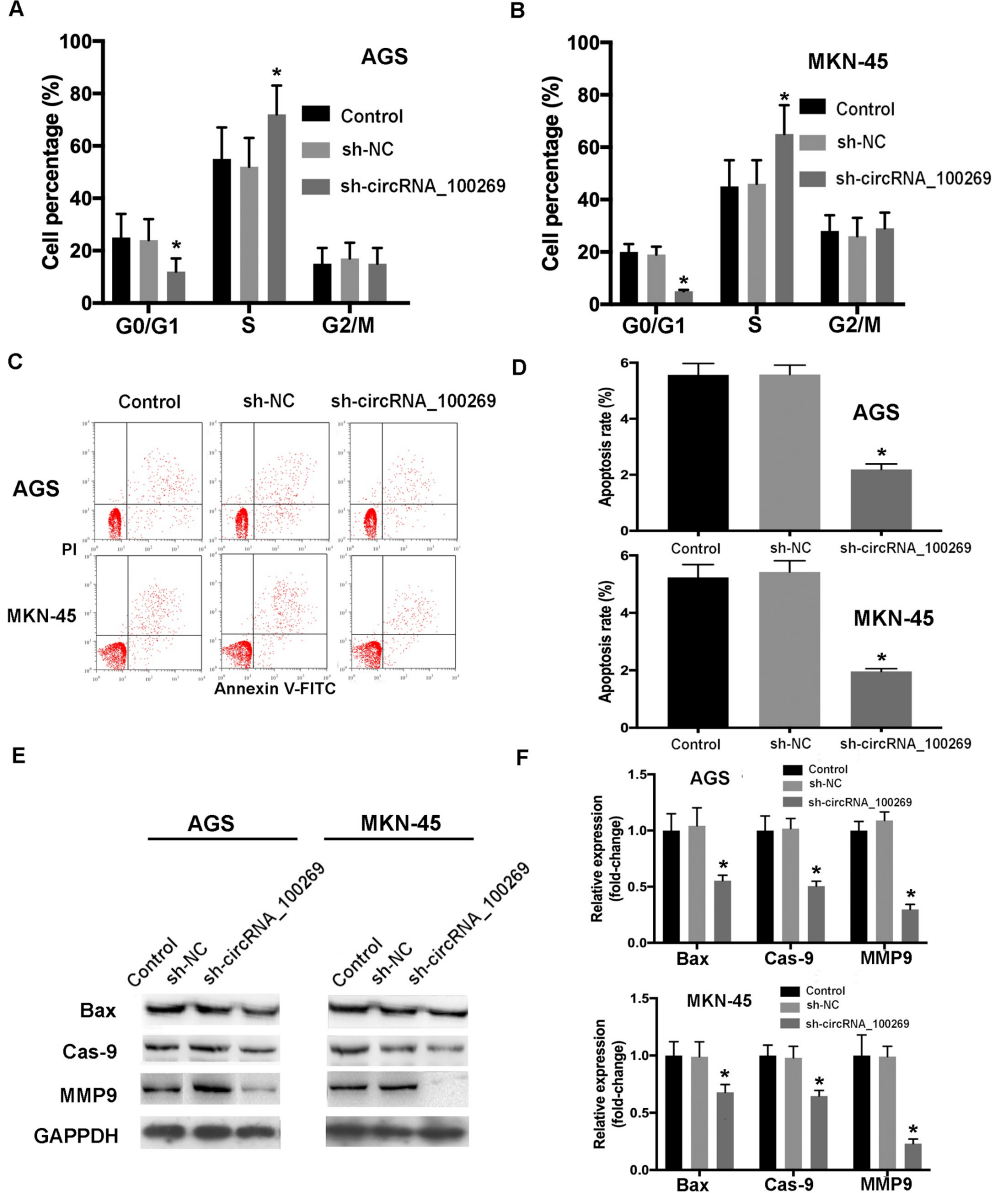
Knockdown of hsa_circRNA_100269 inhibits cell cycle arrest and apoptosis of GC cells. (A and B) The distribution of cell cycle and apoptosis in GC cells transfected with sh-hsa_circRNA_100269 were determined. (C-F) The apoptosis of AGS and MKN-45 cells with hsa_circRNA_100269 knockdown were also examined compared with the controls. All the experiments were performed in triplicate. *P<0.05 vs. non-transfected cells. GC, gastric cancer.

To study the effects of downregulated hsa_circRNA_100269 on cell cycle distribution and apoptosis, further experiments were performed. The results suggested that cell percentage in G0/G1 phase was remarkably reduced, whereas that in S phase was notably increased (Fig 5A and 5B). Additionally, the results of flow cytometry indicated that downregulated hsa_circRNA_100269 expression inhibited the apoptosis of GC cells (Fig 5C and 5D), which was also confirmed by the downregulation of apoptosis-associated markers (Fig 5E and 5F). In summary, these findings revealed that knockdown of hsa_circRNA_100269 could inhibit cell cycle arrest and apoptosis in GC.

### The PI3K/Akt signaling pathway is the novel target of hsa_circRNA_100269 in GC

**Fig 6:**
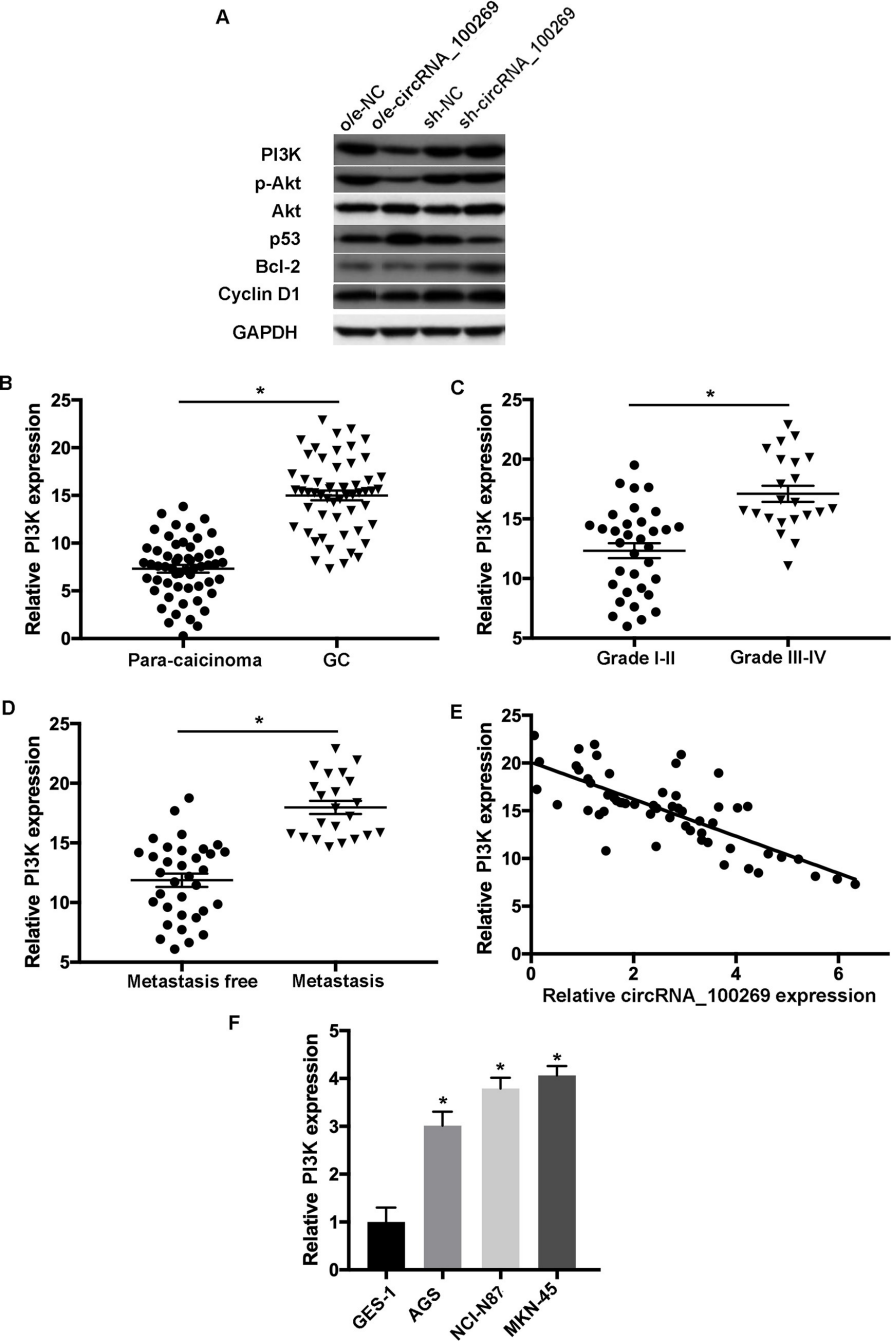
The PI3K/Akt signaling is a promising target of hsa_circRNA_100269 in GC cells. (A) The levels of molecules involved in the PI3K/Akt signaling were assessed in AGS and MKN-45 cells with overexpression/knockdown of hsa_circRNA_100269. (B) The expression of PI3K was examined in GC and matched non-tumour tissues. (C) PI3K expression was evaluated in GC patients with various tumour grades. (D) The expression level of PI3K was determined in GC tissues with metastasis compared to the controls. (E) Spearman’s correlation analysis indicated the inverse correlation between hsa_circRNA_100269 and PI3K in GC samples (r = -0.3291; P = 0.00938). (F) The level of PI3K was also assessed in GC cells compared with normal human gastric epithelial cells. All the experiments were performed in triplicate. *P<0.05 vs. corresponding control group. GC, colorectal cancer.

Further experiments were conducted to investigate the mechanisms of hsa_circRNA_100269-modulated cell growth and apoptosis in GC. Western blot analysis indicated that overexpressed hsa_circRNA_100269 was able to downregulate the expression levels of PI3K and phosphorylated Akt (p-Akt), while knockdown of hsa_circRNA_100269 enhanced the expression of PI3K and p-Akt (Fig 6A). Moreover, the downstream targets of the PI3K/Akt signaling such as p53, Bcl-2 and cyclin D1 were examined. As presented in western blotting, the protein levels of Bcl-2 and cyclin D1 were decreased, whereas p53 was increased in GC cells overexpressing hsa_circRNA_100269, and vice versa. In addition, the levels of PI3K were evaluated in GC and para-carcinoma tissues. The results revealed significant upregulation of PI3K in GC samples compared with normal controls (Fig 6B). Moreover, the expression of PI3K was notably increased in patients with advanced GC (Fig 6C), and the level of PI3K was remarkably upregulated in GC patients with metastasis (Fig 6D). Additionally, the expression levels of PI3K and hsa_circRNA_100269 were inversely correlated (Fig 6E). Furthermore, the level of PI3K was notably elevated in GC cell lines (Fig 6F). Our data indicated that hsa_circRNA_100269 could affect the development of GC by regulating the PI3K/Akt pathway.

### Inactivation of the PI3K/Akt signaling abrogates the biological behavior changes caused by hsa_circRNA_100269 in GC cells

**Fig 7:**
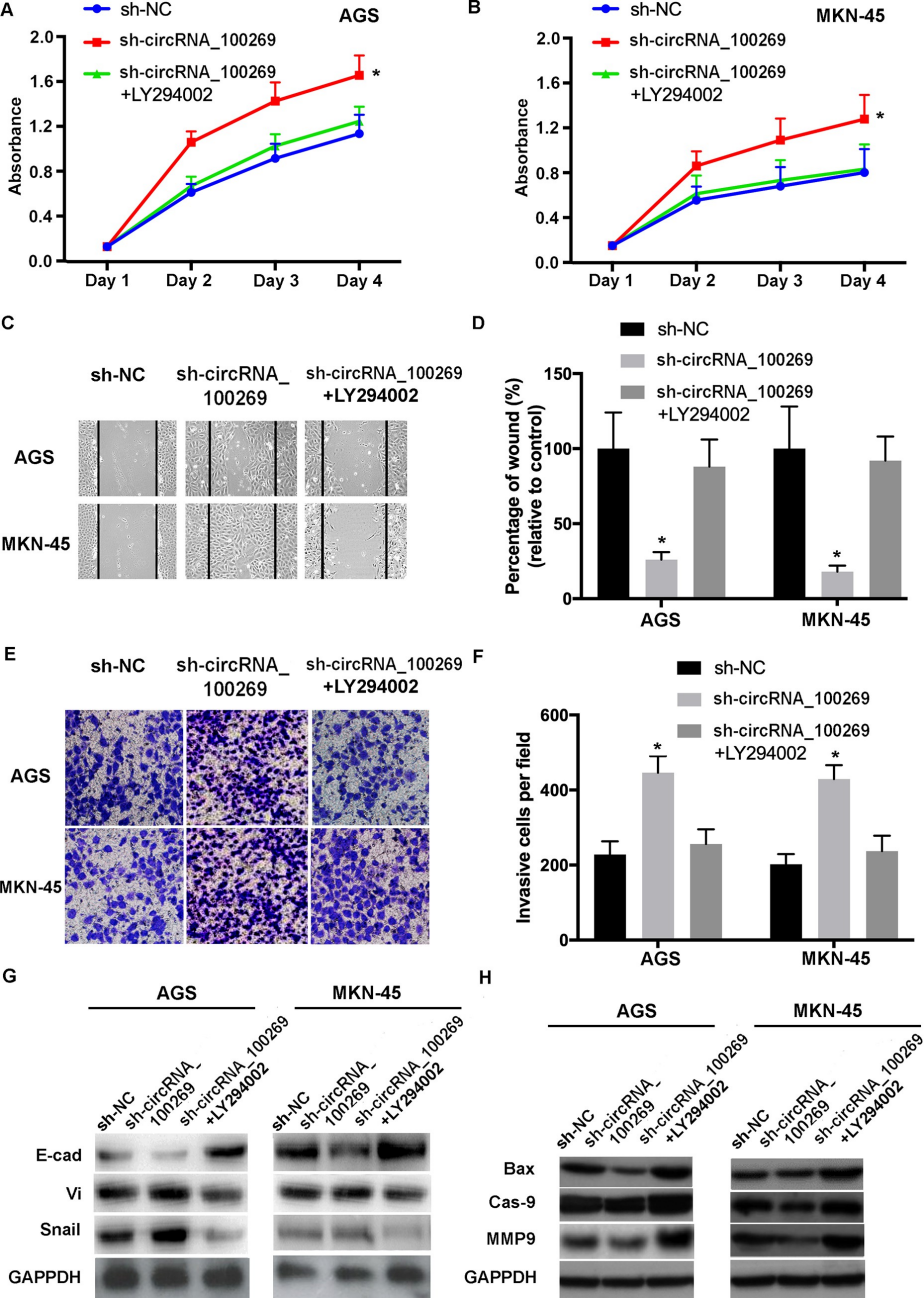
Inactivation of the PI3K/Akt signaling reverses the effects of hsa_circRNA_100269 knockdown in GC cells. (A and B) The proliferation of GC cells transfected with sh-NC, sh-hsa_circRNA_100269 or co-treated with LY294002 was assessed. (C-F) The migration and invasion of treated AGS and MKN-45 cells were determined. (G and H) EMT and apoptosis of transfected GC cells were evaluated. All the experiments were performed in triplicate. *P<0.05 vs. sh-NC. GC, gastric cancer; NC, negative control.

To study whether the influences of hsa_circRNA_100269 on the progression of GC cells was modulated through the PI3K/Akt pathway, GC cells were transfected by sh-NC, sh-hsa_circRNA_100269 or co-treated with LY294002. The results indicated that the effects caused by downregulated hsa_circRNA_100269 in GC cells were abolished by inactivation of the PI3K/Akt signaling (Fig 7). These findings revealed that the PI3K/Akt signaling is involved in hsa_circRNA_100269-mediated cell growth and metastasis in GC, suggesting hsa_circRNA_100269 could suppress the development of GC by suppressing the PI3K/Akt pathway.

### Hsa_circRNA_100269 suppresses the progression of GC in vivo

**Fig 8:**
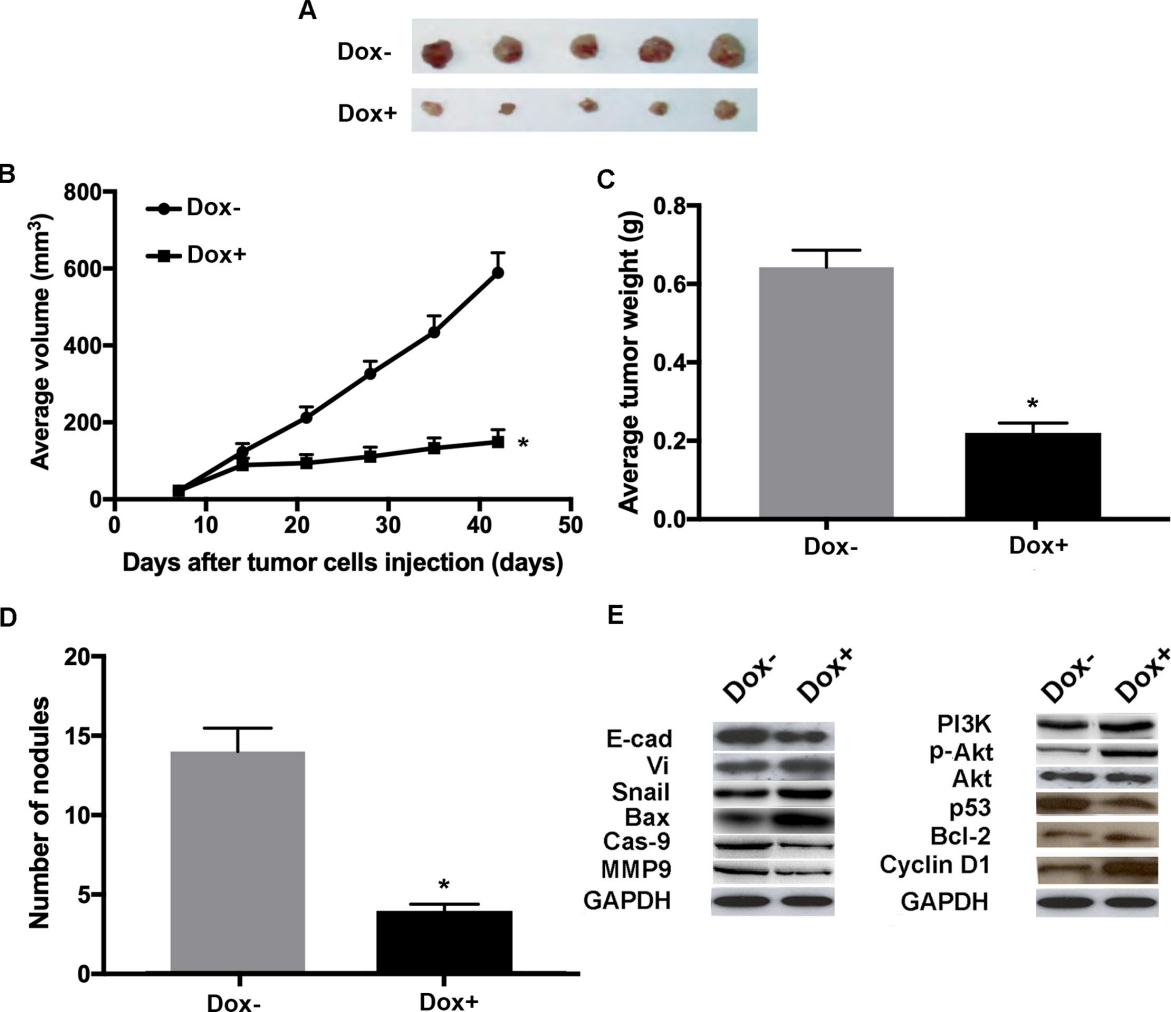
Doxycycline-induced expression of hsa_circRNA_100269 suppress the growth of GC *in vivo*. (A) The tumours in mice were harvested six weeks post-injection. (B) The average tumor volumes were examined in dox-induced group compared with the control. (C) Orthotopic tumor weights at day 42 post-injection were determined. (D) The numbers of nodules in mice were also calculated six weeks post-injection. (E) Protein levels of EMT, apoptosis and PI3K/Akt-associated molecules in xenograft tumors were evaluated by western blot analysis. A total of five mice were included in each experimental group. All the experiments were performed in triplicate. *P<0.05 vs. corresponding control group. n = 5 per each group. GC, gastric cancer.

To further investigate the manipulation of hsa_circRNA_100269 has an effect on tumor growth in vivo, mouse tumor model expressing a dox-inducible form of has_circRNA_100269 was generated. Once the tumor was established, the construct was activated and thereafter the effects of hsa_circRNA_100269 on tumor growth were evaluated. The induction of hsa_circRNA_100269 expression, through doxycycline in drinking water, lead to remarkably smaller tumors (Fig 8A). Examinations on the harvested tumors revealed that the average tumor volume of WT MKN-45 tumors was notably reduced following dox induction (Fig 8B). In addition, the weight of dox-induced tumors was significantly decreased (Fig 8C). Furthermore, the numbers of macroscopic nodules were remarkably reduced in dox+ group (Fig 8D). Moreover, the results of western blot analysis suggested that EMT-, apoptosis- and PI3K/Akt-associated markers exhibited the same expression pattern as observed in the *in vitro* assays following the induced expression of hsa_circRNA_100269 (Fig 8E). The results suggested that hsa_circRNA_100269 could inhibit the development of GC by inactivating the PI3K/Akt signaling *in vivo*.

## Discussion

GC is a common type of malignancy that is a major cause of cancer-related mortality [1,2]. Although the recent treatments of GC have been developed, the therapeutic outcome and prognosis of this disease still remains poor [3–5]. Accumulating evidences have suggested that circRNAs are associated with tumorigenesis of numerous types of cancer [11–13]. Furthermore, aberrant levels of circRNAs have been found in tumour cells, indicating the potential regulatory roles of circRNAs during the development of cancer including GC. Among these previously reported circRNAs, hsa_circRNA_100269 was downregulated in GC, which functions as potential suppressor of tumor progression through regulating miR-630 [15]. Furthermore, miR-630 serves essential roles during EMT via various pathways including the FOXM1 and Slug signaling, subsequently affecting the activity of PI3K/Akt axis [16,17]. However, the exact functions and downstream molecular targets of hsa_circRNA_100269 in GC were not completely understood and require further investigation. Thus, our findings could provide novel insight on the therapeutic development of GC. A previous study has revealed that the activation of PI3K could be triggered by oncogenic factors such as Celastrus orbiculatus extract, consequently resulting in the development of tumour in human osteosarcoma cells [18]. Furthermore, Akt is a downstream molecule of PI3K, which is associated with cell growth and EMT. For instance, activation of the PI3K/GSK-3b/b-catenin signaling promotes the progression and aggressiveness of osteosarcoma [19]. Activated Akt could promote the migration, invasion and EMT of cancer cells, and inhibition of the PI3K/Akt/mTOR signaling could suppress tumour progression [20,21]. Therefore, inactivation of the PI3K/Akt signaling could be a putative therapeutic strategy for cancer treatment.

In this study, the effects of hsa_circRNA_100269-modulated PI3K/Akt axis on the growth and metastasis of GC were elucidated. Our results revealed significant downregulation of hsa_circRNA_100269 in GC patients, whereas the levels of PI3K were remarkably elevated, and their expression were correlated with the tumour grading and occurrence of metastasis. In addition, downregulated hsa_circRNA_100269 and upregulated PI3K were detected in GC cells. Overexpressed hsa_circRNA_100269 notably inhibited the growth, metastasis and EMT of GC cells, and vice versa, knockdown of hsa_circRNA_100269 significantly promoted tumour development both *in vitro*. Furthermore, our data also suggested cell cycle was arrested at G0/G1 phase and cell apoptosis was promoted in GC cells overexpressing hsa_circRNA_100269, where the expression levels of PI3K/Akt-associated molecules were also affected, suggesting the biological behavior changes in GC cells caused by hsa_circRNA_100269 could be dependent on the PI3K/Akt signaling. In addition, hsa_circRNA_100269 was able to inhibit the tumor progression *in vivo*. These data suggested that hsa_circRNA_100269 could inhibit the proliferation, migration and invasion while inducing the cell cycle arrest and apoptosis of GC cells by suppressing the PI3K/Akt signaling pathway. In consistence with our findings, recent studies also reported the regulatory roles of certain circRNAs in GC [22–27]. For example, circNHSL1 and circNF1 could be novel gene regulators in GC, which can affect tumour progression through various signaling pathways including targeting their downstream miRNAs [23,26]. Circ NF1 was able to promote GC development by sponging miR-16 [23]. CircNHSL1 could trigger GC progression via regulating miR-1306-3p/SIX1/vimentin axis [26]. Taken all together, our results revealed the essential roles of the hsa_circRNA_100269/PI3K/Akt signaling on the modulation of GC progression. The detailed mechanisms underlying circRNA_100269-regulated inactivation of PI3K/Akt are unclear. Apart from acting as miRNA sponges [10,15], circRNAs could also exert its regulatory function by regulating transcription and affecting linear mRNA splicing [9,22,24], therefore, the detailed mechanisms require further investigation and should be elucidated in future study. It remains unclear whether circRNA_100269 exert its regulatory roles through acting as miRNA sponge or interacting with target proteins directly, which needs to be further elucidated in future study. For example, bioinformatics tools and databases should be used to explore the potential interactions.

In summary, our results suggested that circRNA_100269 was a promising tumor suppressor that could inhibit the development of GC by targeting the PI3K/Akt pathway. However, there were some limitations in the present study, for instance, to confirm the findings of MTT assay, the expression levels of proliferation-related molecules such as PCNA, Ki-67 could be evaluated in further studies; additionally, to validate the data of Transwell assay, the levels of invasion-associated markers including cathepsin and uPA may also be determined; furthermore, to confirm whether circRNA_100269 is a biomarker for aggressive primary and metastatic GC, the expression profiles of circ_RNA100269 and its downstream molecules should also be evaluated in larger sample size; moreover, the use of an algorithm for subgrouping would be preferable to reduce the bias in the statistics analyses. Our data revealed the key functions of circRNA_100269 and the potential mechanisms underlying the growth and metastasis of GC, which provided substantial evidences on the roles of circRNA_100269 in tumorigenesis. Therefore, circRNA_100269 could be a novel marker for the diagnosis and treatment for GC. At present, inhibition of the PI3K/Akt signaling is considered as a potential therapeutic approach in cancer treatment [28–33]. CircRNA_100269/PI3K/Akt axis could be a putative therapeutic target for the treatment of GC.

## Supporting information

S1 File (PDF)
