# Supplementary figures and images for: Upregulated hsa_circRNA_100269 inhibits the growth and metastasis of gastric cancer through inactivating PI3K/Akt axis
Source: PLoS One. 2021 Apr 26;16(4):e0250603. doi: 10.1371/journal.pone.0250603 (PMC8075232; doi:10.1371/journal.pone.0250603)

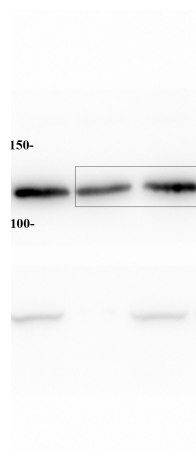

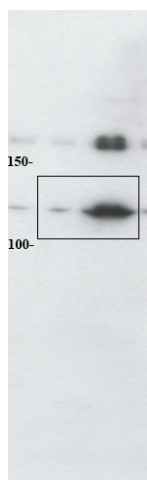

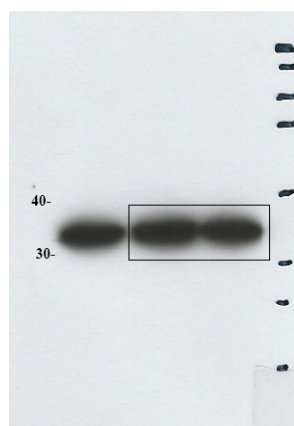

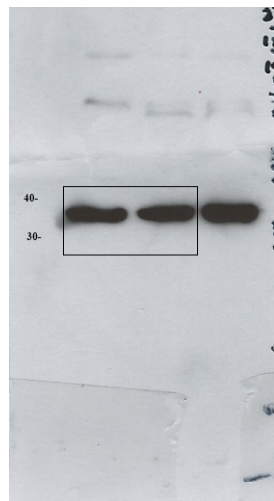

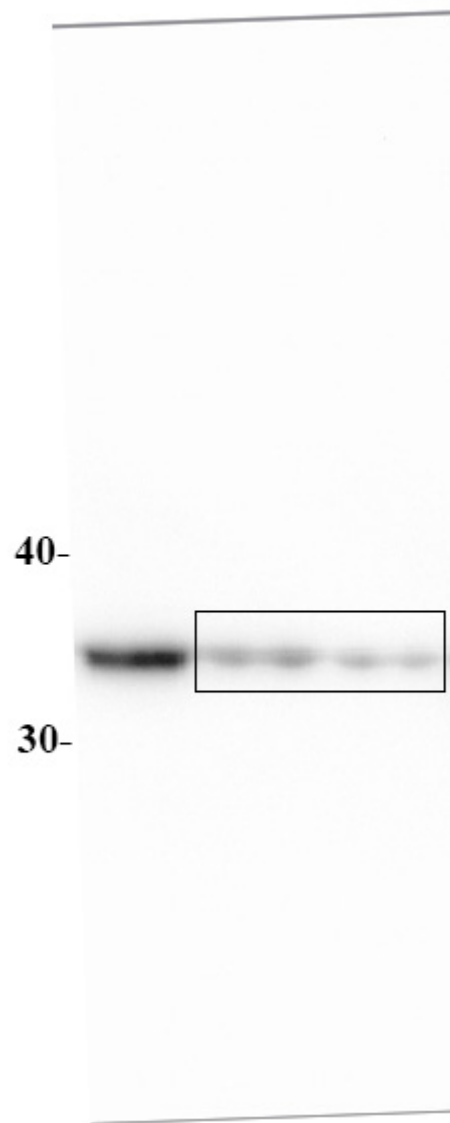

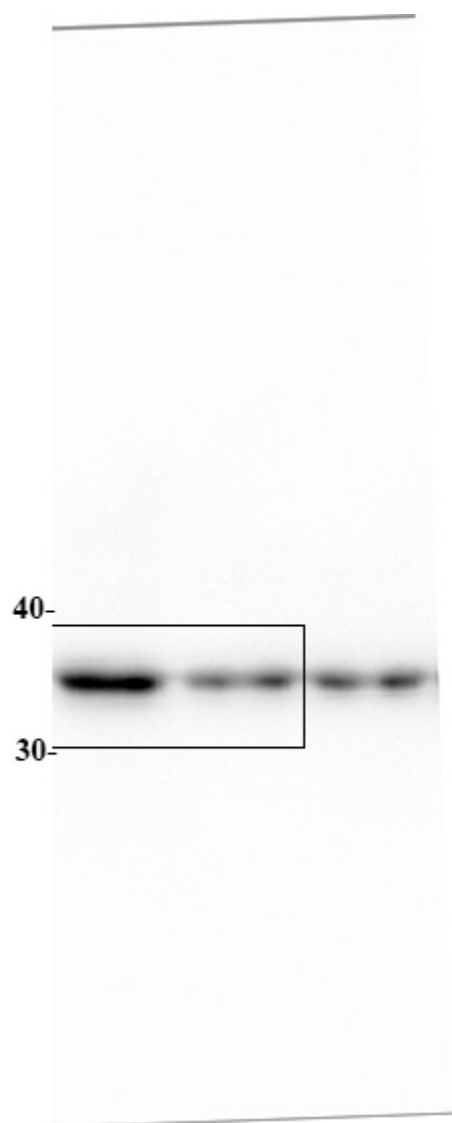

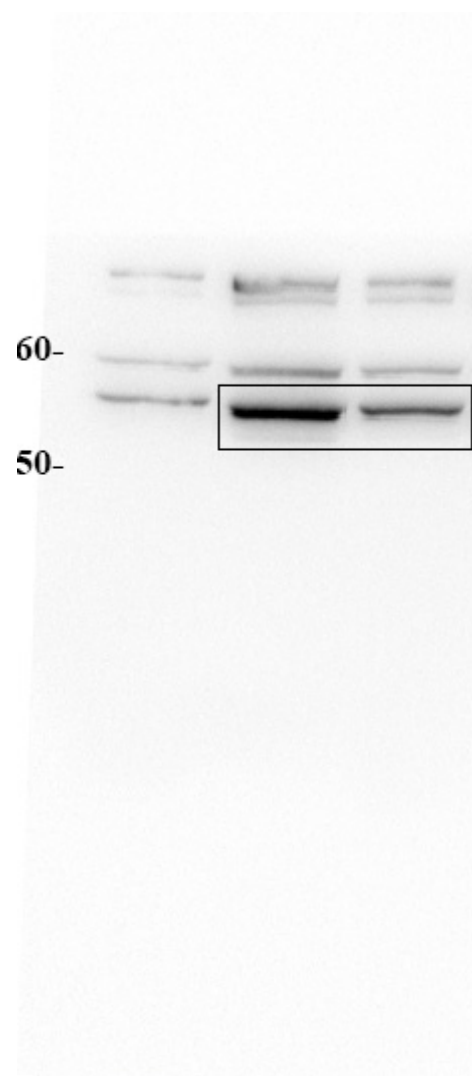

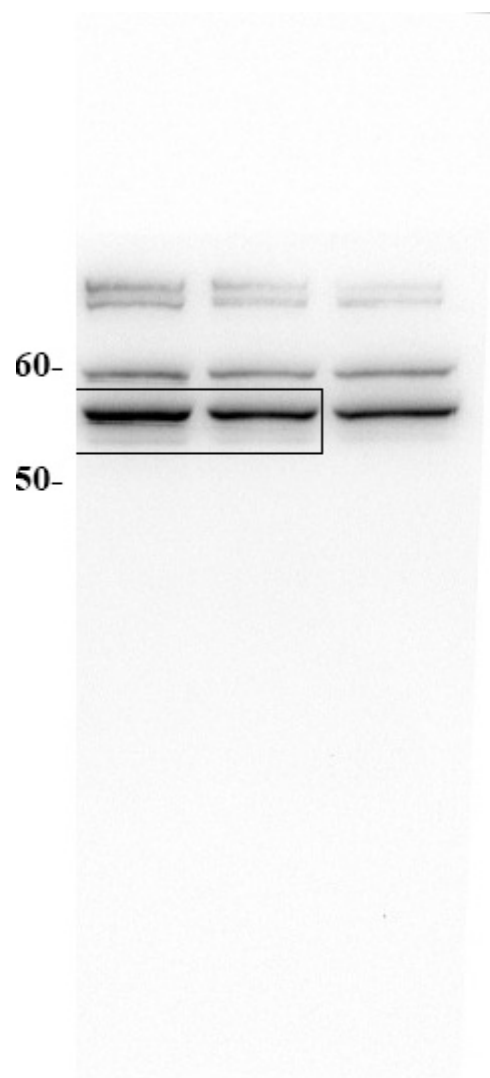

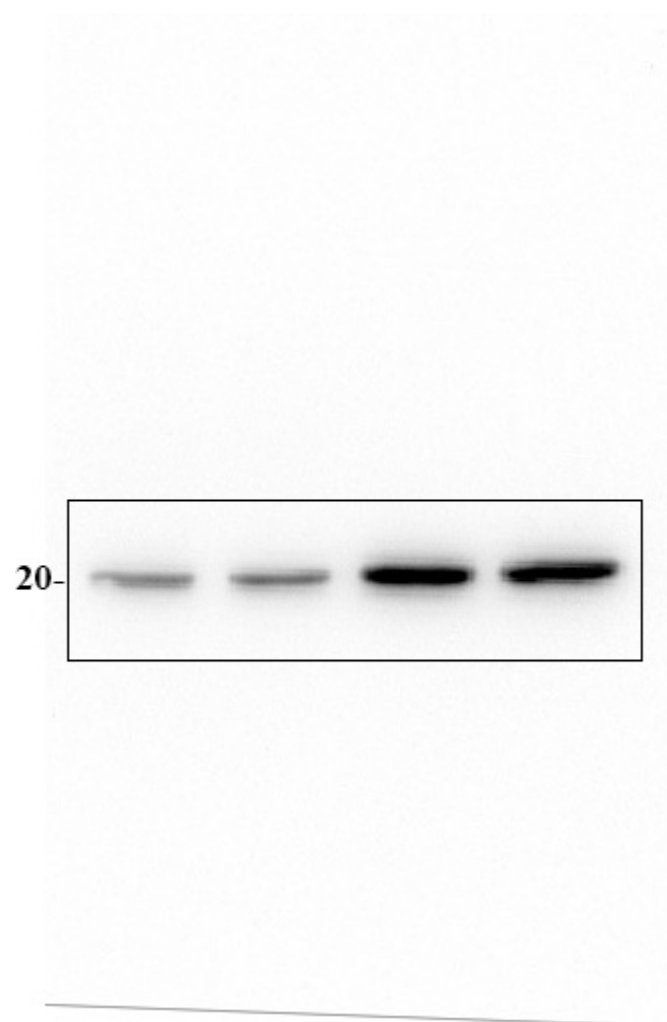

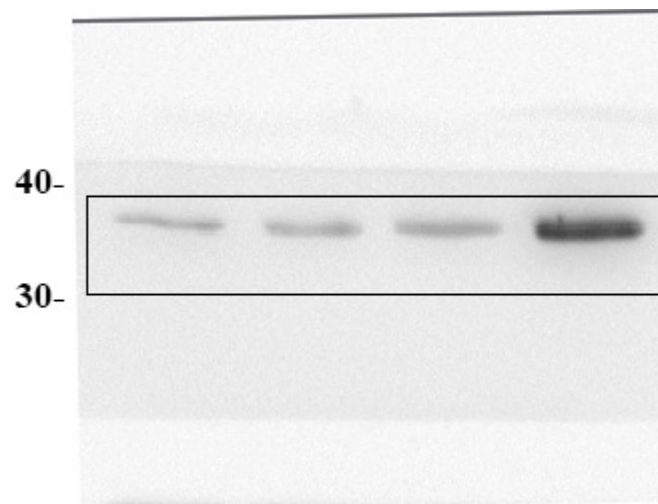

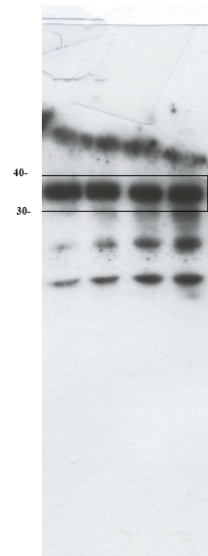

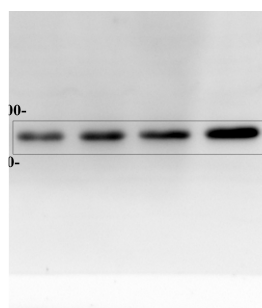

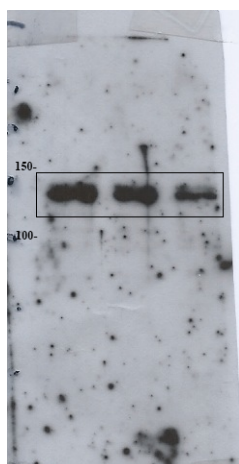

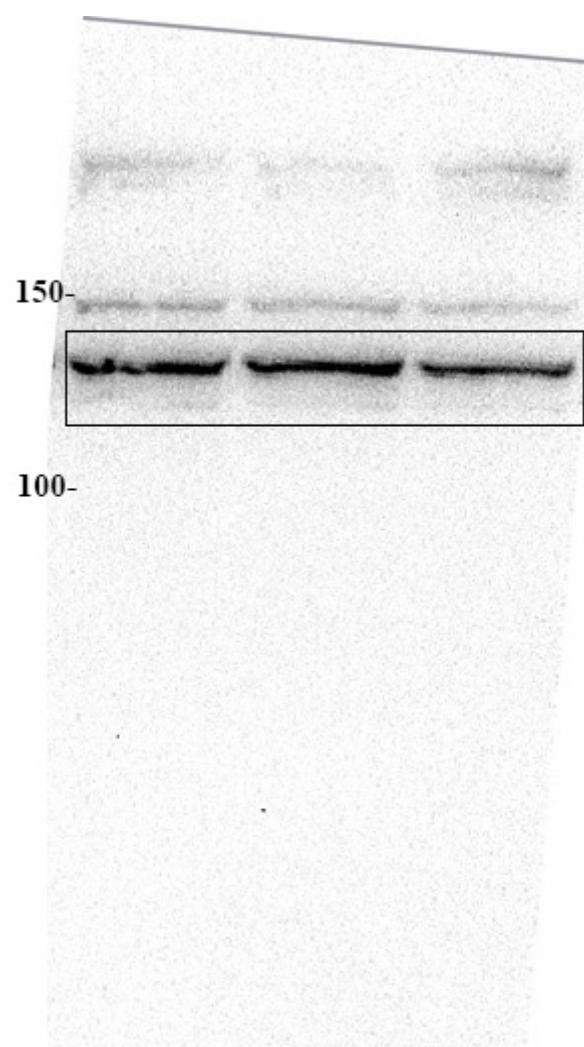

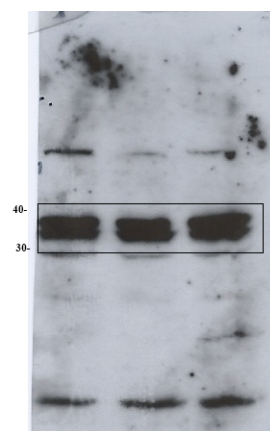

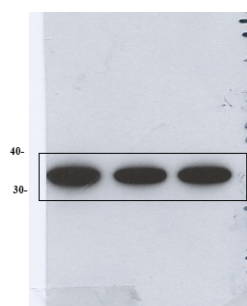

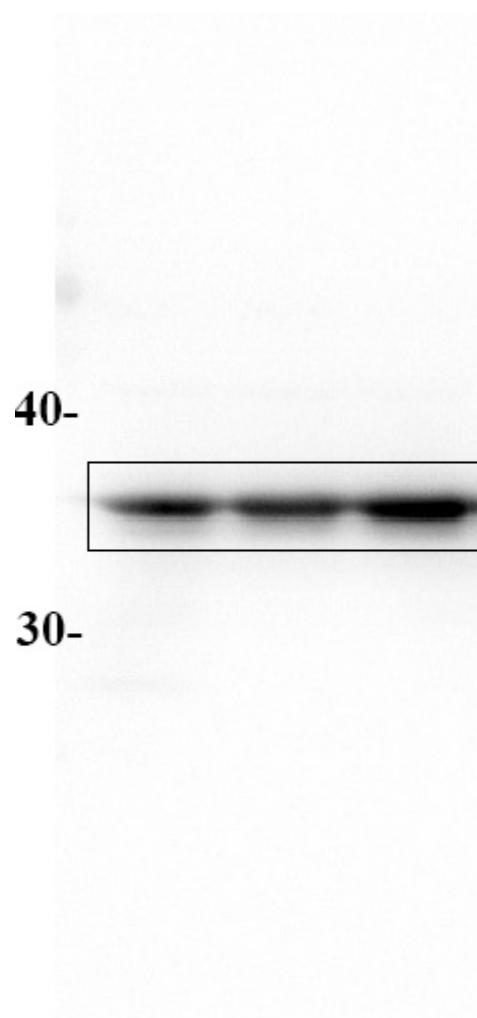

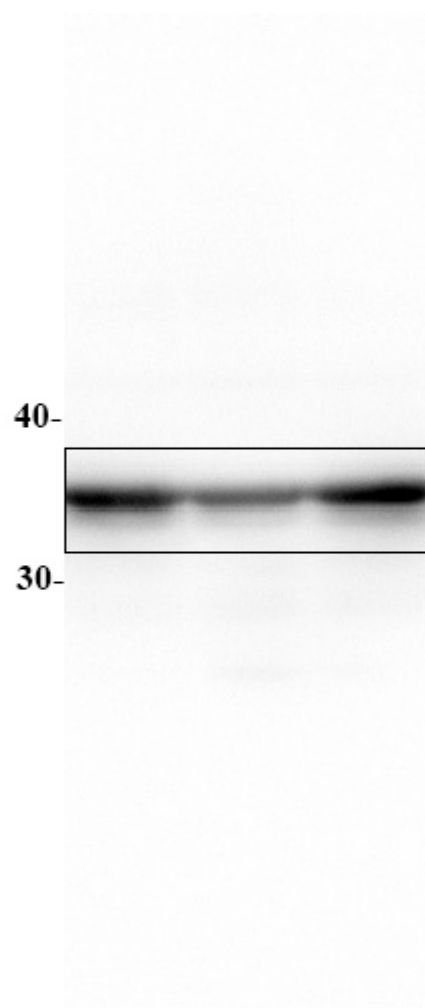

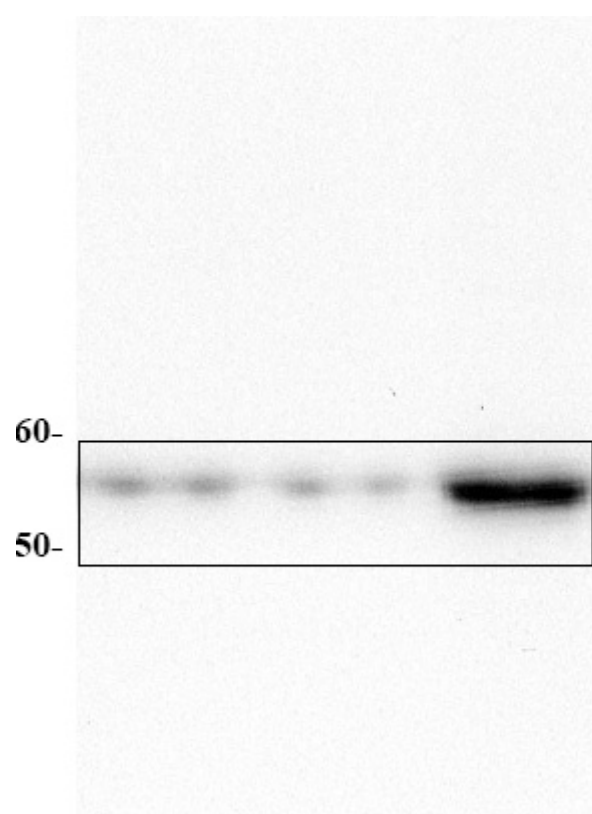

60-

50-

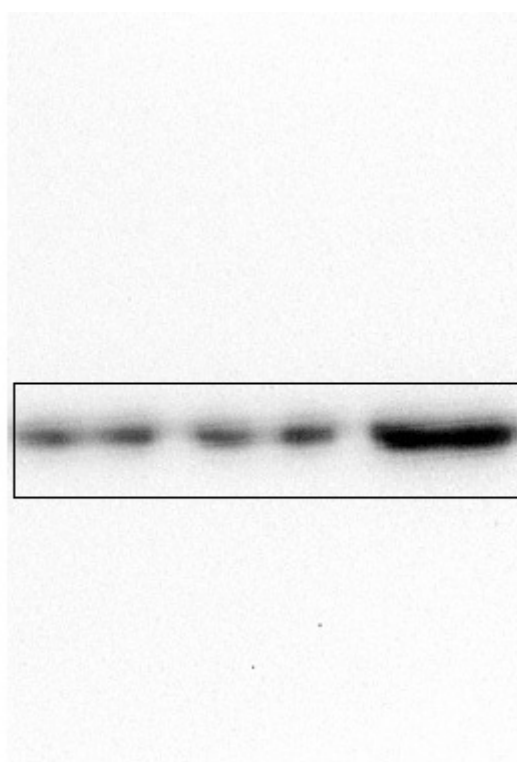

20-

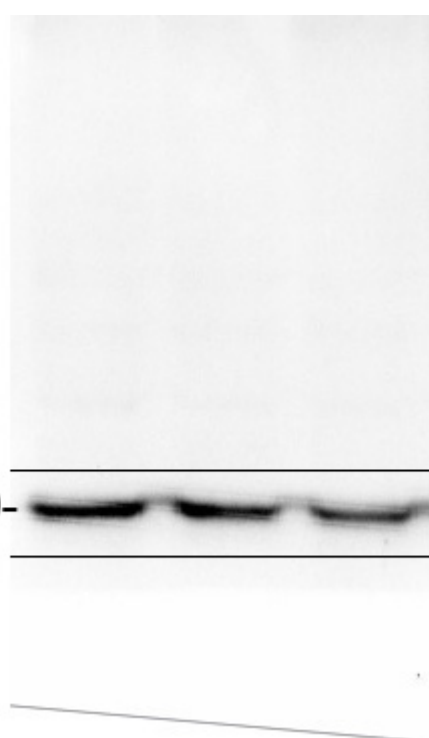

20-

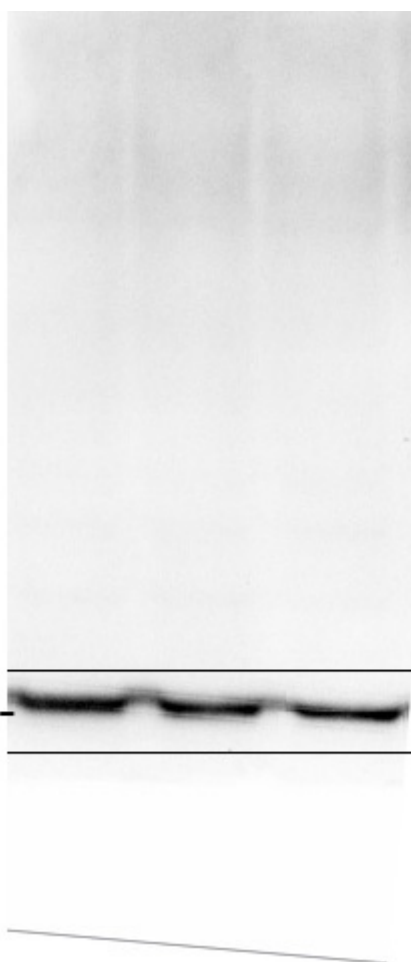

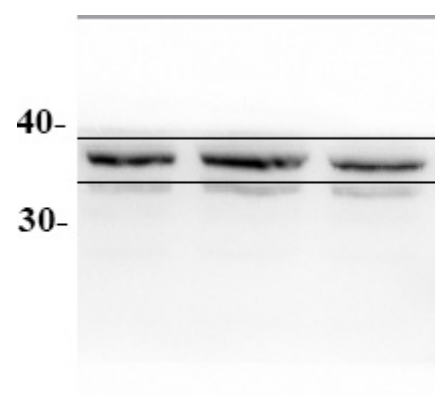

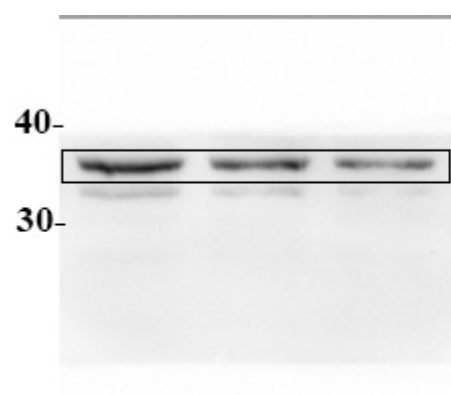

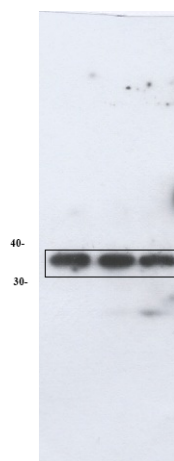

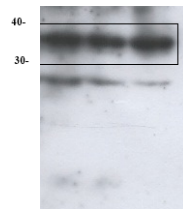

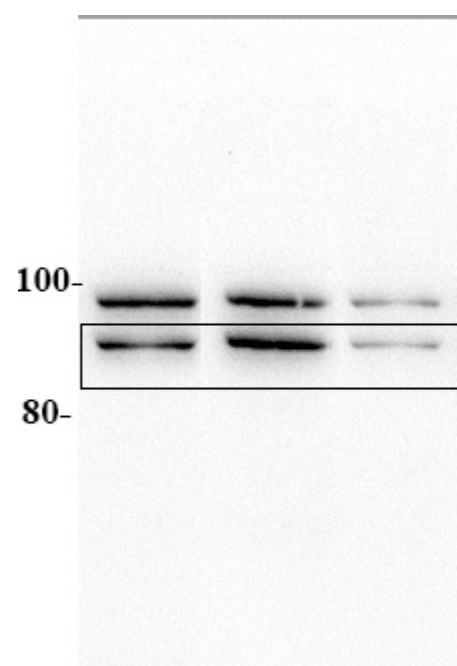

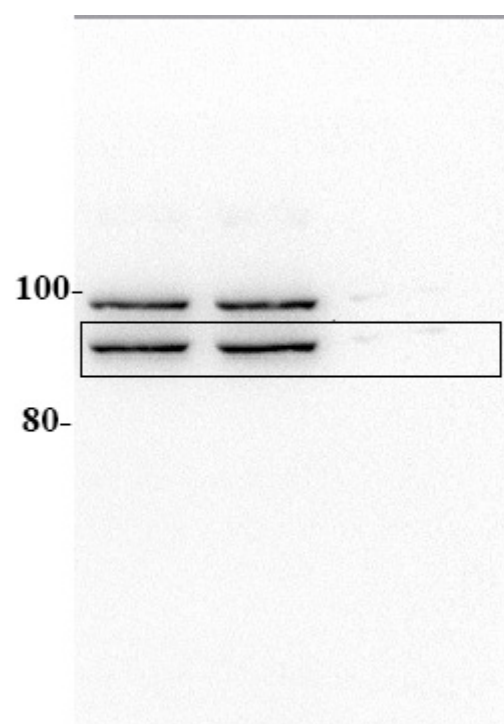

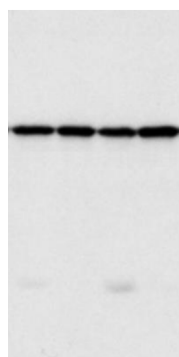

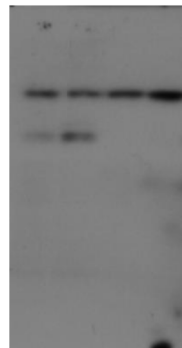

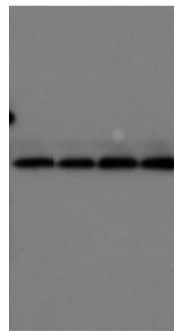

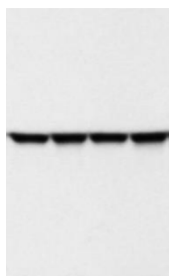

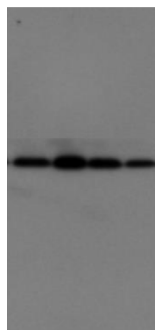

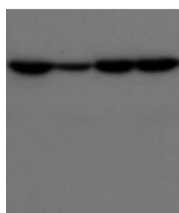

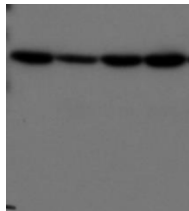

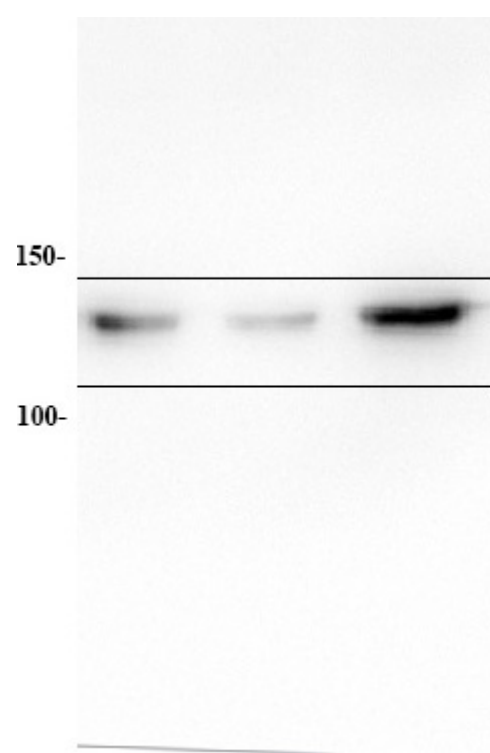

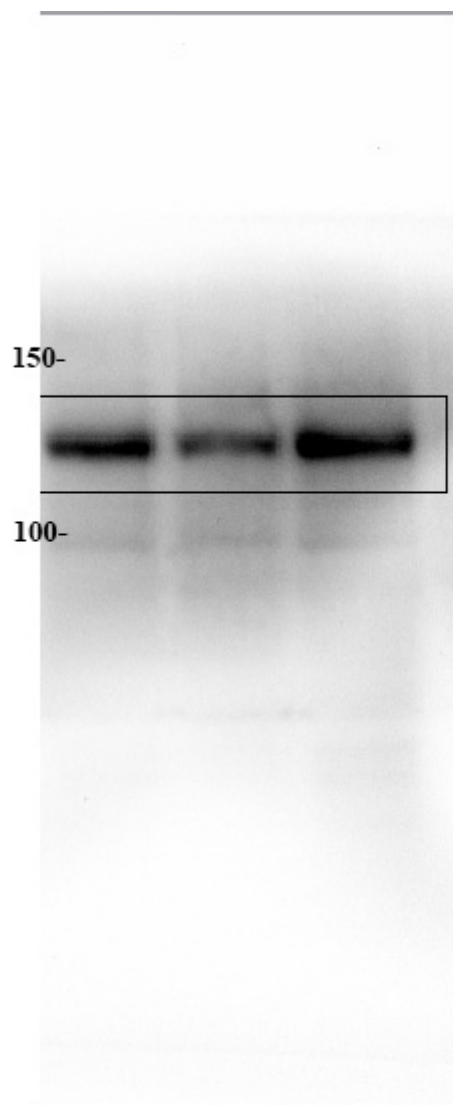

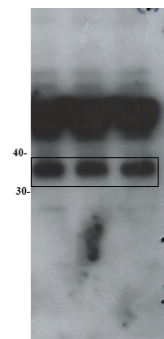

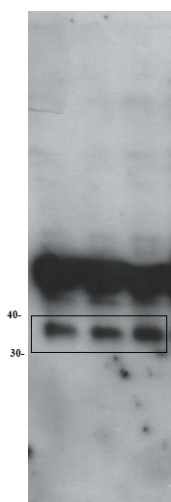

40-

30-

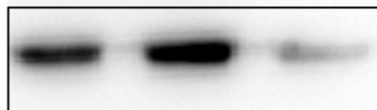

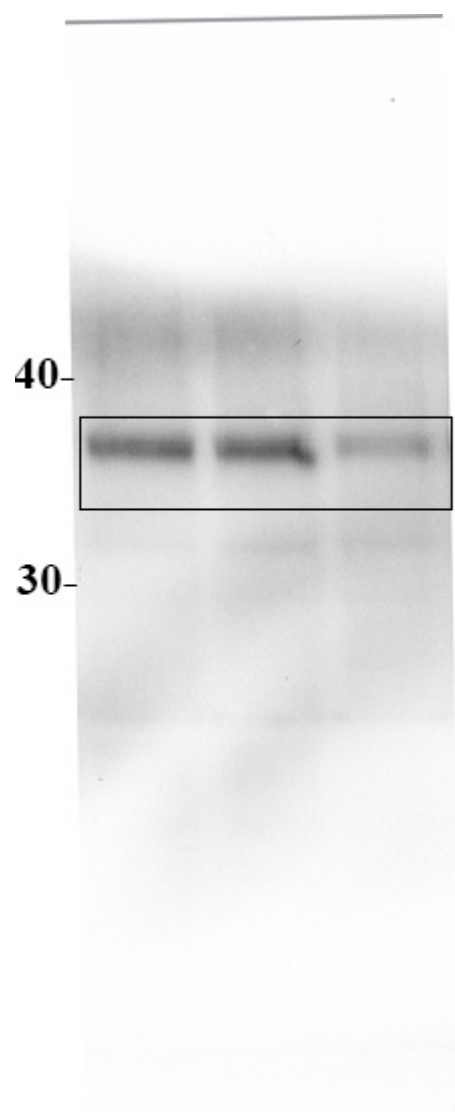

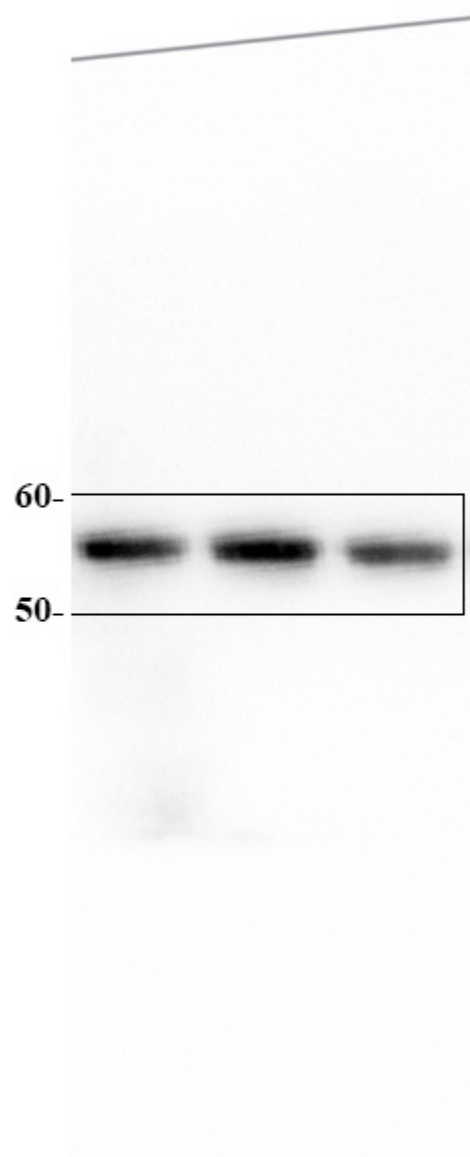

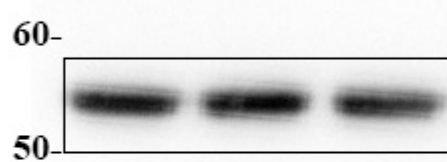

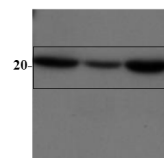

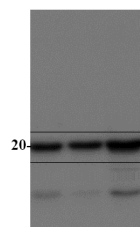

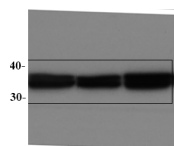

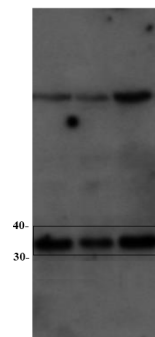

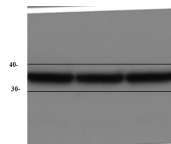

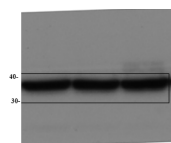

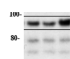

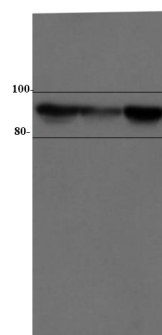

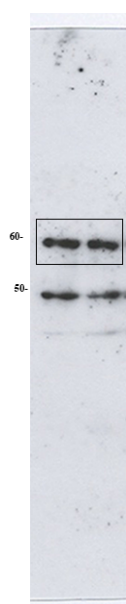

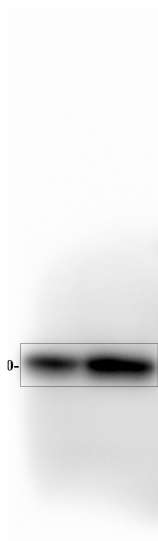

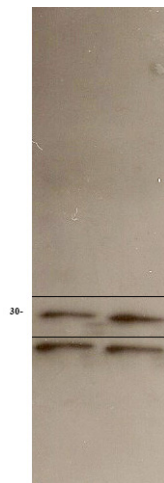

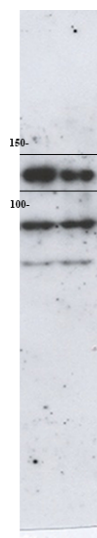

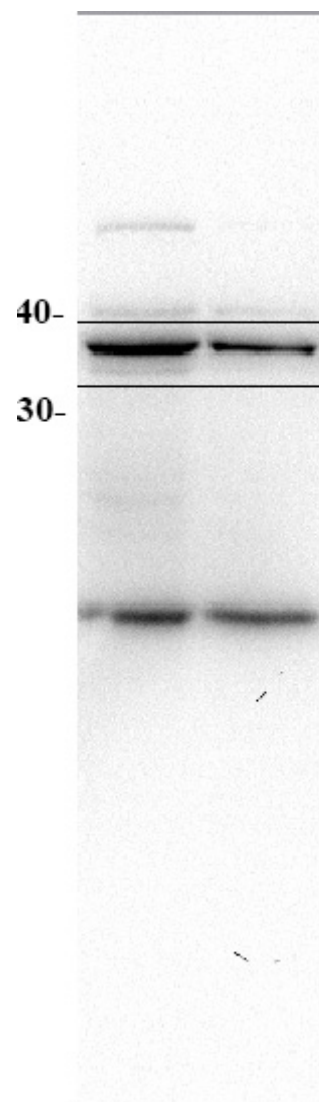

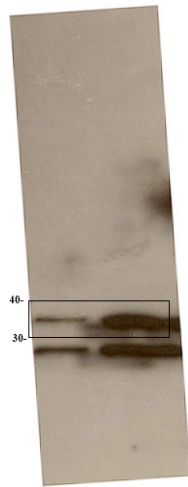

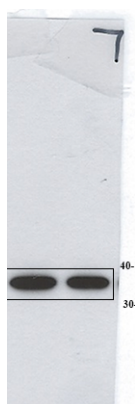

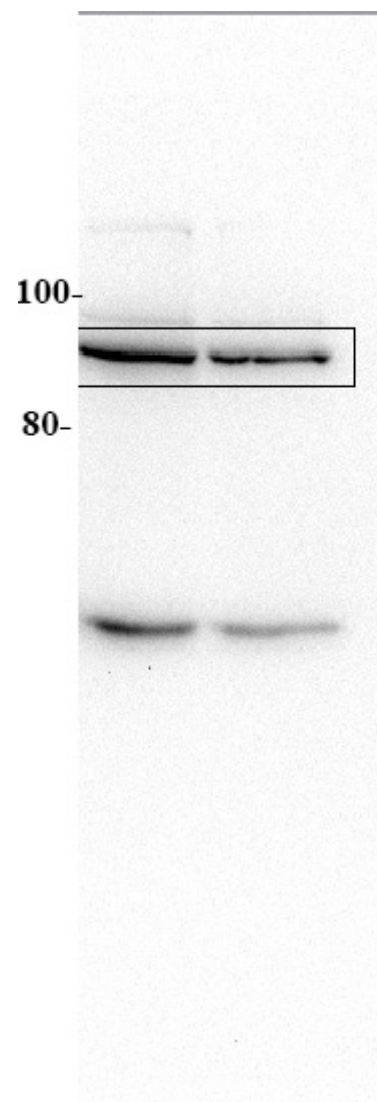

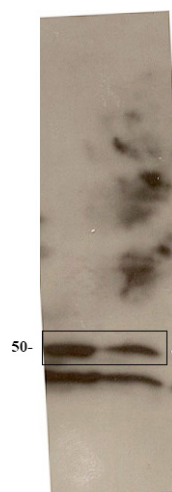

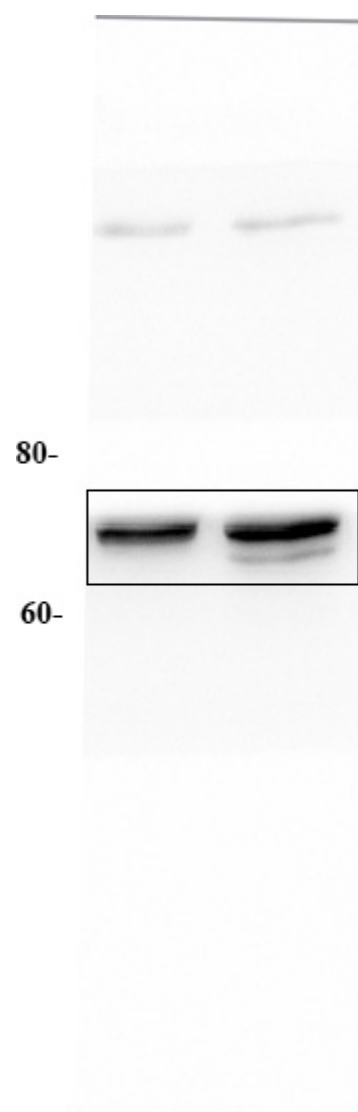

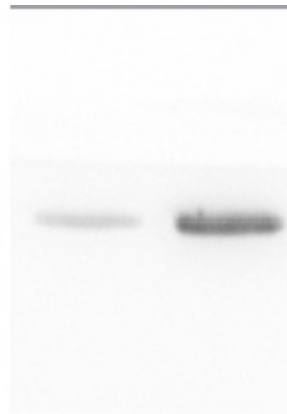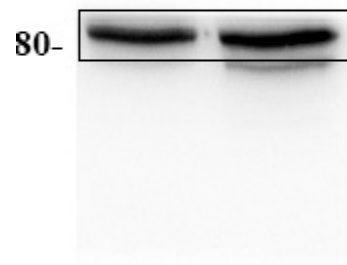

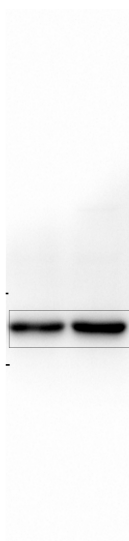

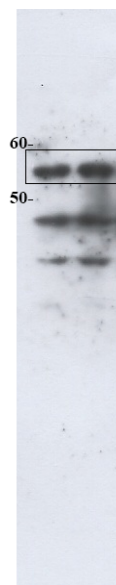

Supplement: S1 File — (PDF) [file pone.0250603.s001.pdf]
